# MiR-483-5p promotes IGF-II transcription and is associated with poor prognosis of hepatocellular carcinoma

**DOI:** 10.18632/oncotarget.21737

**Published:** 2017-10-11

**Authors:** Shaohui Tang, Yanfang Chen, Shufen Feng, Tingzhuang Yi, Xuyou Liu, Qiang Li, Zhilong Liu, Cuiping Zhu, Jianjun Hu, Xi Yu, Min Wang, Guoli Cao, Hui Tang, Caiqun Bie, Feng Ma, Huijun Tang, Gang Du, Jianwei Huang

**Affiliations:** ^1^ Department of Gastroenterology, The First Affiliated Hospital, Jinan University, Guangzhou, Guangdong, China; ^2^ Department of Gastroenterology, Affiliated Hospital of Youjiang Medical University for Nationlities, Baise, Guangxi, China; ^3^ Department of Gastroenterology, The Fifth Affiliated Hospital of Guangzhou Medical University, Guangzhou, Guangdong, China; ^4^ Department of General Surgery, The First Affiliated Hospital, Jinan University, Guangzhou, Guangdong, China; ^5^ Clinical Medicine Research Institute, The First Affiliated Hospital, Jinan University, Guangzhou, Guangdong, China; ^6^ Department of Gastroenterology, The Affiliated Shenzhen Shajing Hospital, Guangzhou Medical University, Shenzhen, Guangdong, China

**Keywords:** miRNAs, transcription regulation, ago proteins, liver cancer, inferior clinical outcome

## Abstract

The human insulin-like growth factor-II (IGF-II) gene transcribes four mRNAs (P1 mRNA-P4 mRNA), and P3 mRNA overexpression contributes to hepatocarcinogenesis. IGF-II-derived miR-483-5p is implicated in the development of cancers. Here, we investigated the involvement of miR-483-5p in P3 mRNA overexpression regulation and its role in hepatocellular carcinoma. Our results showed that miR-483-5p up-regulated P3 mRNA transcription by targeting the 5′-untranslated region (5′UTR) of P3 mRNA in hepatocellular carcinoma. The mechanism was involved in recruiting of an argonaute 1(Ago1)-argonaute 2 (Ago2) complex to the P3 mRNA 5′UTR and the P3 promoter of IGF-II gene by miR-483-5p, accompanied by increased enrichment of RNA polymerase II and activating histone marks histone 3 lysine 4 trimethylation (H3K4me3), histone 3 lysine 27 acetylation (H3K27ac), and histone 4 lysine 5/8/12/16 acetylation (H4Kac) at the P3 promoter. High miR-483-5p expression was an independent predictor for shorter survival of HCC patients. The findings suggest that miR-483-5p promotes P3 mRNA transcription by recruiting the Ago1-Ago2 complex to the P3 mRNA 5′UTR and is associated with poor prognosis of HCC. Our results display a potential new model for miRNAs to up-regulate gene expression.

## INTRODUCTION

The human insulin-like growth factor-II (IGF-II) is a fetal growth factor that is involved in fetal growth and development [1]. The gene encoding IGF-II contains nine exons and four promoters (P1–P4), and each of these promoters initiates distinct transcription, yielding a promoter-specific transcript (P1 mRNA-P4 mRNA) that has a unique 5′-untranslated region (5′UTR) and an identical 3′UTR [2]. IGF-II is expressed predominantly in fetal liver under physiological conditions through the activation of P2-P4 promoters; however, its expression is significantly down-regulated shortly after birth because of remarkable decrease or loss of the activity of P2–P4 promoters [3]. There is compelling clinical and experimental evidence that IGF-II is involved in hepatocarcinogenesis [4]. Indeed, IGF-II expression is elevated in human hepatocellular carcinoma (HCC) tissues [5], HCC cell lines [6, 7], and HCC animal models [8]. Increased level of IGF-II in HCC results mainly from the transcriptional up-regulation of P3 mRNA and P4 mRNA [9, 10]. However, the reasons and mechanisms responsible for the reactivation of the two fetal transcripts are not well clarified.

MicroRNAs (miRNAs) are a class of small endogenous non-coding RNAs (21–23 nucleotides) that posttranscriptionally down-regulate gene expression by inhibiting translation or inducing mRNA degradation through complementary binding to the 3′UTR of target messenger RNA (mRNA) [11]. However, several studies have showed that miRNAs can also bind the 5′UTR of target mRNAs resulting in translation up-regulation [12, 13], which overturns the inhibitory effect of miRNAs on gene expression.

Studies have indicated that miRNAs may be involved in IGF-II expression regulation in human cancers. Lu et al. showed that methylated let-7a-3 was associated with low IGF-II expression and favorable prognosis of ovarian cancer [14], and that let-7a could increase IGF-II expression in cancer cells [15]. Because P3 mRNA is the most abundant transcript of IGF-II gene [9, 10] and the regulation mechanism of its overexpression has not yet been well studied in HCC, the present study was done to explore the involvement of miR-483-5p, an intronic miRNA of IGF-II gene [16], in P3 mRNA overexpression regulation and its role in HCC. Our results may provide novel target molecules for targeted therapy of HCC.

## RESULTS

### miR-483-5p is up-regulated and associates with P3 mRNA expression of the IGF-II gene in human HCC cells

**Figure 1:**
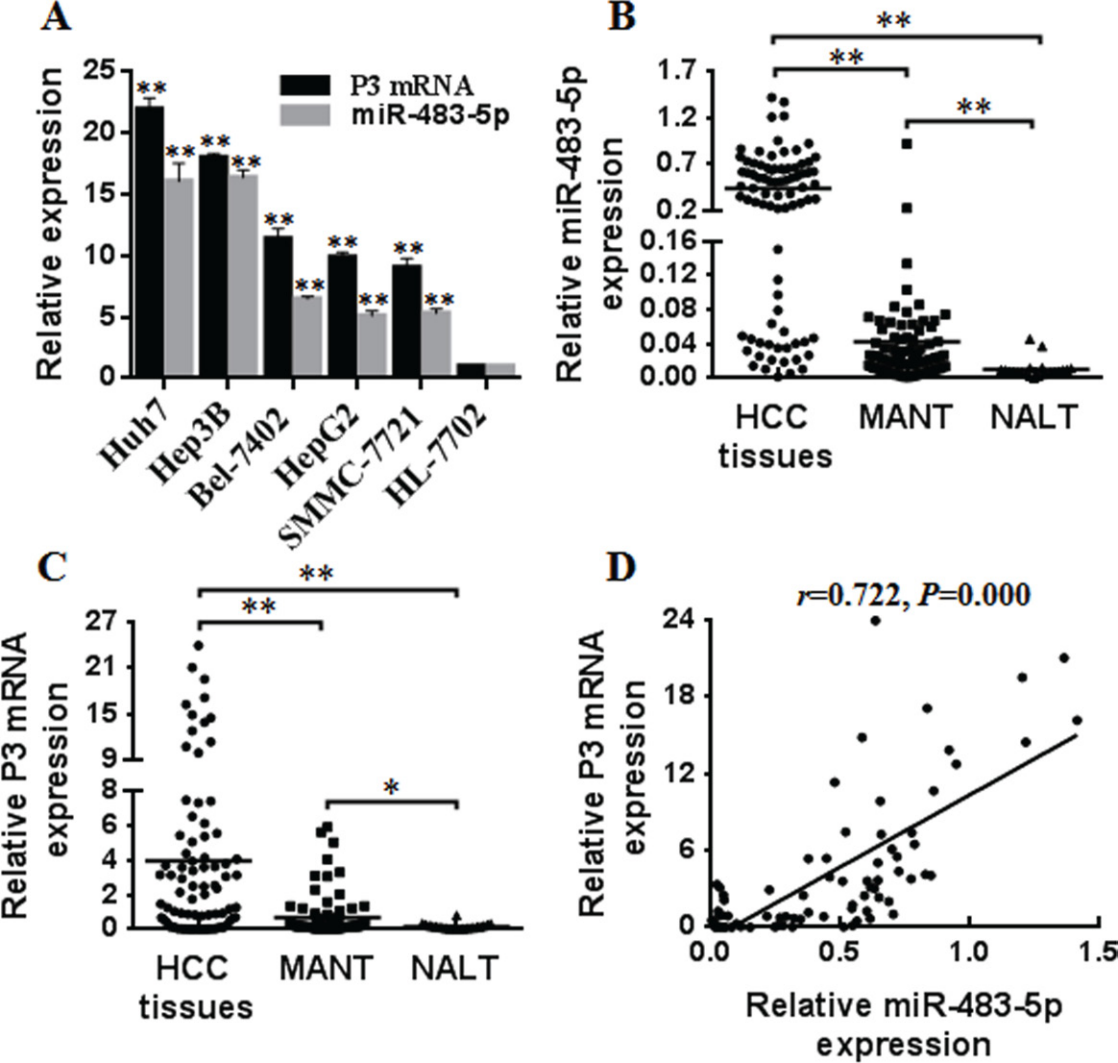
The overexpression of miR-483-5p and P3 mRNA in HCC cells and human HCC tissues (**A**) The expression of miR-483-5p and P3 mRNA was detected in HCC cells (Huh7, Hep3B, Bel-7402, HepG2, SMMC-7721) and the normal human liver cell line (HL-7702) by qRT-PCR. ^**^P < 0.01 versus HL-7702 cells. (**B** and **C**) The expression of miR-483-5p and P3 mRNA was detected in 83 human HCC tissues, 83 MANT, and 22 NALT by qRT-PCR. ^*^P < 0.05 versus NALT, ^**^P < 0.01 versus MANT or NALT. (**D**) P3 mRNA abundance was positively correlated with miR-483-5p expression in HCC tissues (r = 0.722, *P* = 0.000). HCC, hepatocellular carcinoma; MANT, matched adjacent nontumorous tissues; NALT, normal adult liver tissues.

We and other researchers have showed that P3 mRNA of IGF-II gene is the most abundant transcript in HCC [9, 10, 17, 18], which becomes the major contributor of IGF-II overexpression. To investigate that if miRNAs are involved in increased P3 mRNA expression in HCC, we predicted candidate miRNAs targeting the P3 mRNA 5′UTR (NM_000612) only, that is to say, they do not bind to the 3′UTR and 5′UTR of the other three transcrips, P1-P4 promoters, and IGF-II protein coding region of IGF-II gene using RegRNA (http://regrna.mbc.nctu. edu.tw/html/prediction.html). We identified 70 miRNA candidates (Supplementary Table 1) and the top five miRNAs (miR-1915, miR-658, miR-1268, miR-483-5p, and miR-1182) with a remarkable minimal free energy value were selected for further validation. qRT-PCR analysis showed that only miR-483-5p expression was up-regulated in human HCC cell lines (Huh7, Hep3B, Bel-7402, HepG2, SMMC-7721) compared with the normal human liver cell line (HL-7702), and the expression level of P3 mRNA and miR-483-5p was positively correlated in the above HCC cell lines (r = 0.960, *P* = 0.01) (Figure 1A). The findings suggest that miR-483-5p is up-regulated and associates with P3 mRNA expression of IGF-II gene in human HCC cells.

### miR-483-5p is overexpressed and linked with P3 mRNA expression in human HCC tissues

To further investigate the potential involvement of miR-483-5p in HCC and its association with P3 mRNA expression, we examined the expression of miR-483-5p and P3 mRNA in 83 human HCC tissues, 83 matched adjacent nontumorous tissues (MANT), and 22 normal adult liver tissues (NALT). The results revealed that the expression level of both miR-483-5p and P3 mRNA was higher in HCC tissues than that in MANT and NALT, and the expression of miR-483-5p and P3 mRNA was also higher in MANT than that in NALT (Figure 1B and 1C). Further linear correlation analysis showed that P3 mRNA expression level was positively correlated to miR-483-5p expression in HCC tissues (r = 0.722, *P* = 0.000) (Figure 1D). These data suggest that miR-483-5p overexpression is involved in HCC development and linked with P3 mRNA expression at tissue level.

### miR-483-5p up-regulates P3 mRNA transcription by targeting the P3 mRNA 5′UTR of IGF-II gene

**Figure 2:**
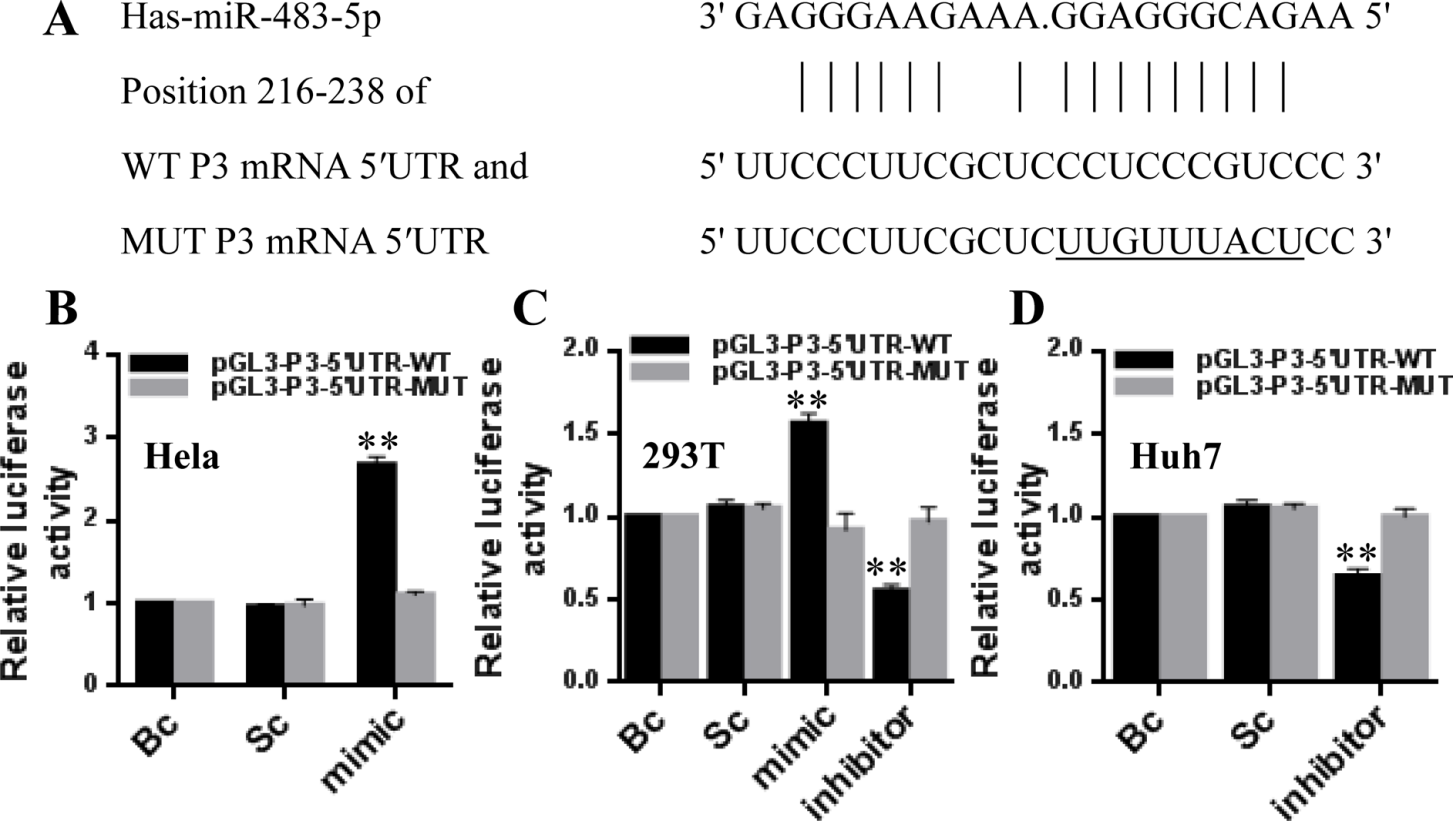
miR-483-5p directly recognizes the 5′UTR of P3 mRNA (**A**) The sequence of human miR-483-5p and the putative human P3 mRNA 5′UTR fragment containing wild-type (WT) or mutant (MUT) miR-483-5p-binding sequence. (**B–D**) Dual-luciferase assay in HeLa, 293T, and Huh7 cells co-transfected with the firefly luciferase constructs containing the WT or MUT 5′UTR of P3 mRNA and miR-483-5p mimic, miR-483-5p inhibitor or scrambled control. ^**^P < 0.01 versus scrambled or blank control. Bc, blank control; Sc, scrambled control; mimic, miR-483-5p mimic; inhibitor, miR-483-5p inhibitor.

To confirm whether miR-483-5p directly recognizes the P3 mRNA 5′UTR, a human full-length 5′UTR of P3 mRNA (NM_000612) containing wild-type or mutant miR-483-5p binding sequence (Figure 2A) was cloned into the pGL3 promoter vectors, and these recombinant vectors were termed pGL3-P3-5′UTR-WT and pGL3-P3-5′UTR-MUT reporters, respectively. The reporters were co-transfected into HeLa cells (a low expression of miR-483-5p) with miR-483-5p mimic or scrambled control (Dharmacon), 293T cells (a medium expression of miR-483-5p) with miR-483-5p mimic, miR-483-5p inhibitor or scrambled control, and Huh7 cells (a high expression of miR-483-5p) with miR-483-5p inhibitor or scrambled control. In comparison with scrambled control or blank control (untreated group), miR-483-5p mimic induced about a 2.7-fold (HeLa) or 1.6-fold (293T) increase in the relative luciferase activity of the pGL3-P3-5′UTR-WT reporter, whereas in the pGL3-P3-5′UTR-MUT reporter, the relative luciferase activity was unaffected by miR-483-5p mimic (Figure 2B and 2C). Conversely, miR-483-5p inhibitor induced about a 45% (293T) or 36% (Huh7) decrease in the relative luciferase activity of the pGL3-P3-5′UTR-WT reporter, but the relative luciferase activity of the pGL3-P3-5′UTR-MUT reporter was unaffected by miR-483-5p inhibitor (Figure 2C and 2D). The findings indicate that miR-483-5p promotes gene expression via miR-483-5p binding sequence located at the 5′UTR of P3 mRNA.

**Figure 3:**
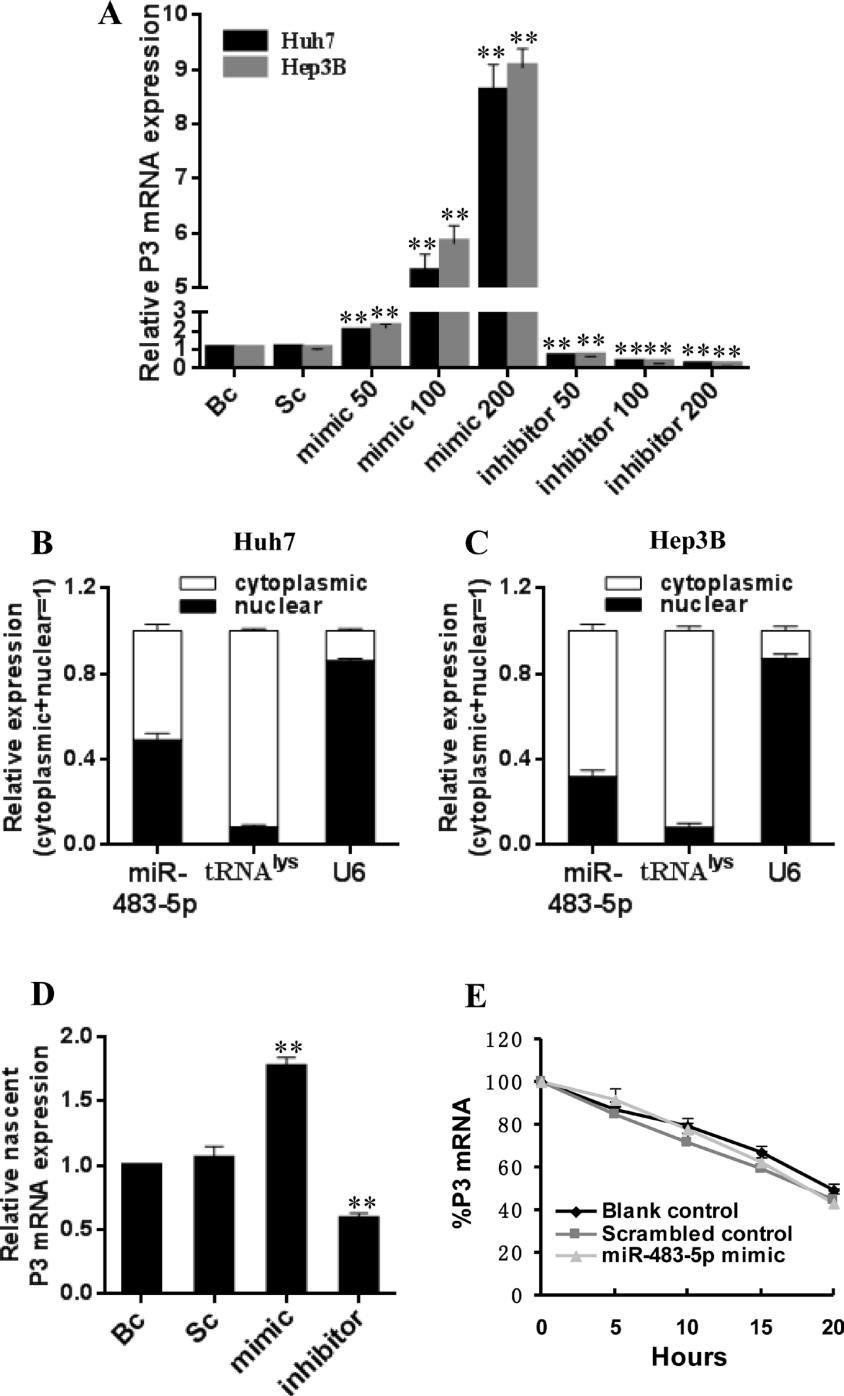
Effect of miR-483-5p on P3 mRNA transcription in Huh7 and Hep3B cells (**A** and **B**) Subcellular fractionation location of miR-483-5p. miR-483-5p expression level in cell cytoplasm or nucleus of Huh7 and Hep3B cells was detected by qRT-PCR. Lysine-tRNA (tRNA^lys^) was used as a cytosol marker and U6 snRNA was used as a nucleus marker. (**C**) miR-483-5p mimic or inhibitor promoted or inhibited P3 mRNA expression in Huh7 and Hep3B cells in a dose-dependent manner (a range of concentrations of 50 nM –200 nM), respectively. ^**^P < 0.01 versus scrambled or blank control. (**D**) miR-483-5p mimic or inhibitor promoted or inhibited nascent P3 mRNA expression in Huh7 cells, respectively, as measured by nuclear run-on assays. ^**^P < 0.01 versus scrambled or blank control. (**E**) Effect of miR-483-5p on P3 mRNA stability was analyzed by the use of transcriptional inhibitor actinomycin D in Huh7 cells, and stability of P3 mRNA was not affected by miR-483-5p. miR-483-5p mimic group versus scrambled or blank control group indicated *P* > 0.05 at various time points post-treatment. Bc, blank control; Sc, scrambled control; mimic, miR-483-5p mimic; inhibitor, miR-483-5p inhibitor.

To further validate that if miR-483-5p can increase P3 mRNA expression, we transfected miR-483-5p mimic, miR-483 inhibitor, and scrambled control into Huh7 and Hep3B cells. We found that the ectopic overexpression of miR-483-5p increased the expression of P3 mRNA in a dose-dependent manner, whereas the inhibition of endogenous miR-483-5p expression resulted in down-regulation of P3 mRNA level in a dose-dependent manner compared with scrambled or blank control in the two cell lines (Figure 3A). However, transfection of miR-483-5p mimic, miR-483 inhibitor, and scrambled control had no significant effect on the levels of P1 mRNA, P2 mRNA, and P4 mRNA of IGF-II gene (data not shown). The induction of IGF-II protein expression by miR-483-5p was further confirmed by Western blot analysis in the two cell lines (Supplementary Figure 1A–1C). The above results indicate that miR-483-5p promotes IGF-II expression only through up-regulating P3 mRNA level.

To evaluate whether increased P3 mRNA level is due to elevated transcription by miR-483-5p, the following assays were performed. We first examined miR-483-5p expression in the nuclear and cytoplasmic fractions of HCC cell lines Hep3B and Huh7, and the results showed that miR-483-5p expression was detected in both nucleus and cytoplasm (Figure 3B and 3C). These data indicate that mature miR-483-5p trafficks to the nucleus in the above cell lines. Our results are similar to previous reports that showed that a fraction of cellular miRNAs was located in the nucleus [19–21]. Then, a nuclear run-on assay was performed. The ectopic overexpression of miR-483-5p induced nascent P3 mRNA expression, whereas the inhibition of endogenous miR-483-5p expression caused ascent P3 mRNA down-regulation compared with scrambled and blank controls in Huh7 cells (Figure 3D). Finally, in order to determine whether increased P3 mRNA expression might be attributed to stabilization by miR-483-5p, the stability of P3 mRNA was evaluated using the transcriptional inhibitor actinomycin D. No significant difference was found in the stability of P3 mRNA in Huh7 cells transfected with miR-483-5p mimic or scrambled control, or in untreated Huh7 cells (blank control) (Figure 3E), suggesting that miR-483-5p may not increase P3 mRNA stability. Taken together, these results suggest that miR-483-5p may promote P3 mRNA transcription by targeting the P3 mRNA 5′UTR.

### Transcriptional up-regulation of P3 mRNA by miR-483-5p involves the RNAi factors, Ago1 and Ago2

**Figure 4:**
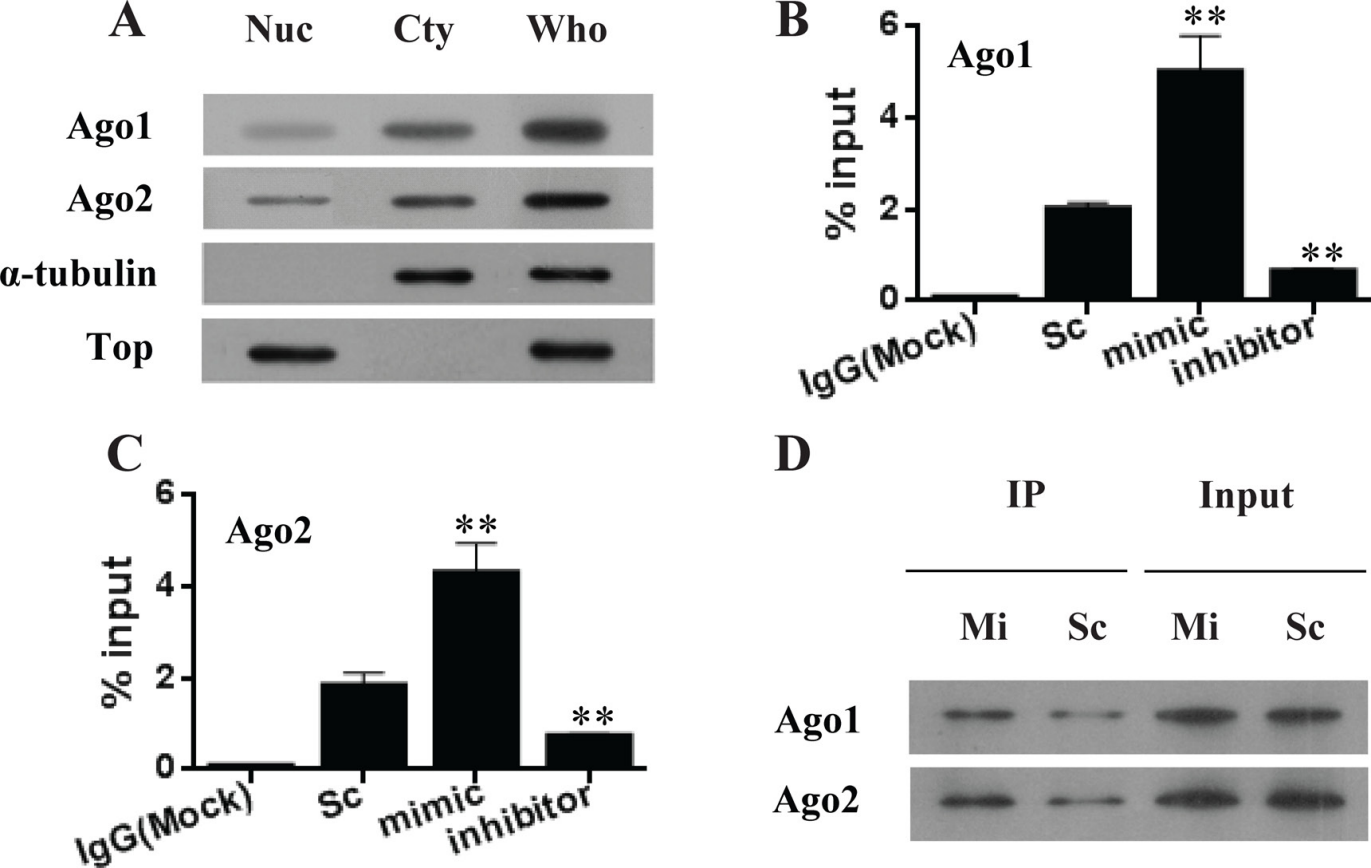
miR-483-5p increases the recruitment of endogenous Ago 1 and Ago 2 to its complementary target site of the P3 mRNA 5′UTR (**A**) Identification of Ago 1 and Ago 2 proteins in the nuclei of Huh7 cells. α-tubulin is a cytoplasmic marker, and topoisomerase I is a nuclear marker. (**B** and **C**) RIP analysis of Ago1 and Ago2 recruitment to the 5′UTR of P3 mRNA by miR-483-5p. ^**^P < 0.01 versus scrambled control. (**D**) Biotinylated miR-483-5p mimic associates with Ago1 and Ago2. Immunoprecipitation (IP) was performed with Dynabeads M-280 Streptavidin, followed by immunoblotting with anti-Ago1 or anti-Ago2 antibody. Nuc, nuclear extracts; Cty, cytoplasmic extracts; Who, whole-cell extracts; Top, topoisomerase I; Sc, scrambled control; mimic and Mi, miR-483-5p mimic; inhibitor, miR-483-5p inhibitor.

It has been shown that mature miRNAs are bound by argonaute (Ago) proteins and loaded into the RNA-induced silence complexes (RISCs) to inhibit gene expression [22]. The key RNA interfering (RNAi) components such as Ago proteins have been found in the nucleus [23, 24]. In the present study, both endogenous Ago1 and Ago2 were found to be present in the nuclei of Huh7 cells (Figure 4A). To investigate if the transcriptional activation of P3 mRNA by miR-483-5p is associated with Ago1 and Ago2 in the nucleus, RIP assays were performed using nuclear extracts of Huh7 cells. The results showed that miR-483-5p mimic increased the recruitment of Ago1 and Ago2 to the 5′UTR of P3 mRNA (spanning the binding site of miR-483-5p), whereas miR-483-5p inhibitor decreased the association of Ago1 and Ago2 with the 5′UTR of P3 mRNA compared with scrambled control (Figure 4B and 4C). Additionally, no association was found between Ago1 or Ago2 and the 3′UTR of P3 mRNA under any treatment condition (data not shown). To examine whether miR-483-5p interacts with Ago1 and Ago2, a ChIP–immunoblot analysis was performed. The result indicated that miR-483-5p was bound to Ago1 and Ago2, suggesting that miR-483-5p directly interacts with Ago1 and Ago2 (Figure 4D). The findings suggest that miR-483-5p directs Ago1 and Ago2 in a sequence-specific manner to its complementary target site within the 5′UTR of the P3 mRNA, and that Ago1 and Ago2 is involved in transcriptional activation of P3 mRNA by miR-483-5p.

### miR-483-5p increases the association of endogenous Ago1 and Ago2 with the P3 promoter of IGF-II gene

**Figure 5:**
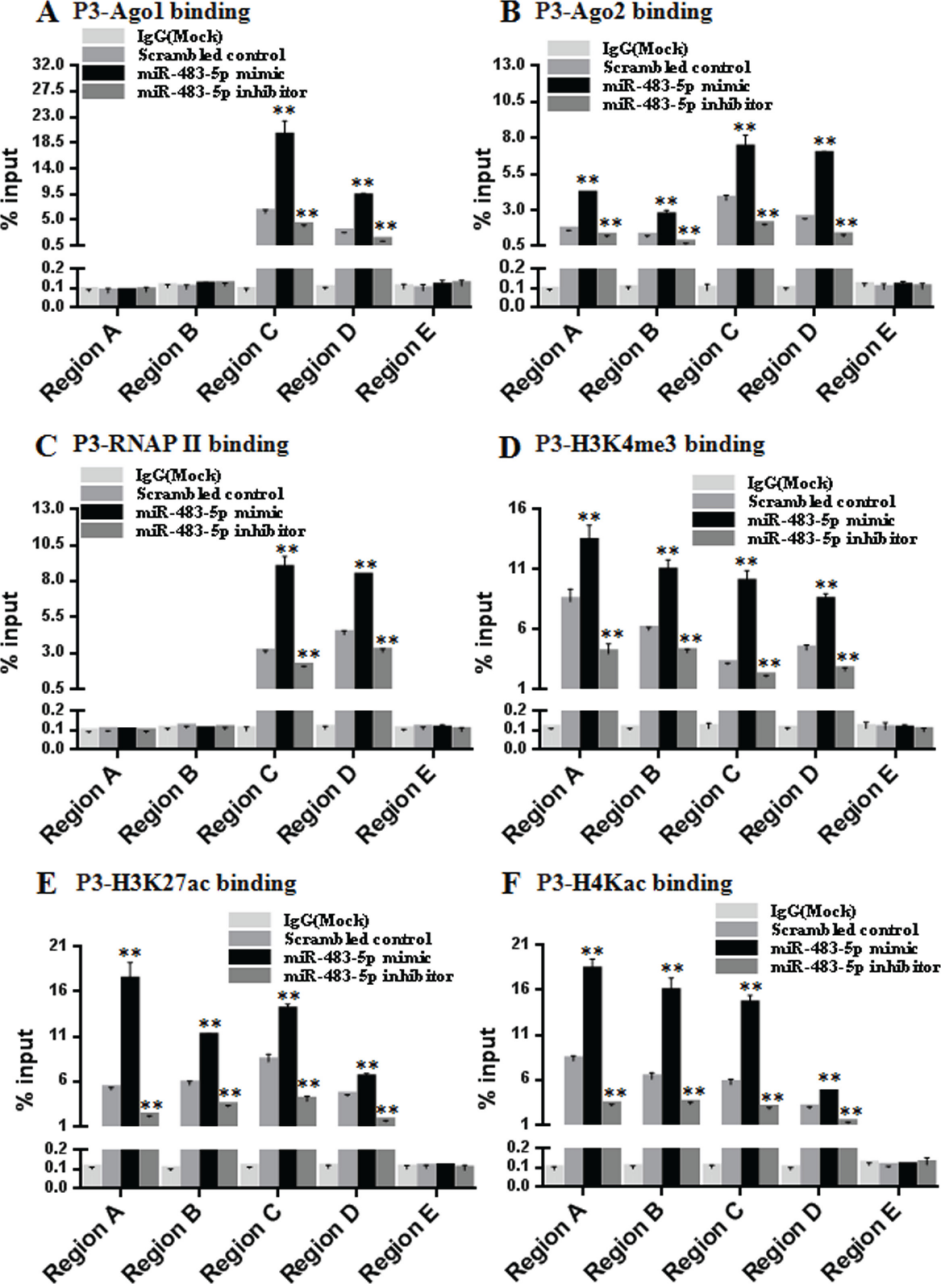
miR-483-5p increases enrichment of endogenous Ago1 and Ago2, RNA polymerase II, and activating histone marks at the P3 promoter (**A–F**) The chromatin from Huh7 cells transfected with miR-483-5p mimic, miR-483-5p inhibitor or scrambled control was isolated and immunoprecipitated with antibodies specific for anti-Ago1 (A), anti-Ago2 (B), anti-RNAP II) (C), anti-H3K4me3 (D), anti- H3K27ac (E), and anti-H4Kac (F). Associated DNA was analyzed by qPCR using five primer pairs covering the Region A(–841/–529), B(–729/–271), C(–354/+122), D(+137/+459), and E (+368/+788) that amplify the extended P3 promoter region (–841/+788). ^**^P < 0.01 versus scrambled control.

Ago1 and Ago2 have been found to associate with gene promoters [25, 26]. Since Ago1 and Ago2 are related to P3 mRNA transcription activation, we reasoned that they may be recruited to the P3 promoter of IGF-II gene. To test this hypothesis, we performed ChIP assays to examine the association of endogenous Ago1 and Ago2 with the P3 promoter in Huh7 cells. Five primer pairs covering the regions (Region A-E) from the -841 bp upstream of the transcription start site to +788 bp downstream of the P3 promoter were designed. ChIP assays revealed that miR-483-5p mimic or miR-483-5p inhibitor treatment significantly increased or decreased enrichment of Ago1 in Region C (–354/+122) and D (+137/+459), and increased or decreased enrichment of Ago2 in Region A (-841/-529), B (–729/–271), C, and D, respectively. Furthermore, no significant enrichment of Ago1 or Ago2 was detected in Region E (+368/+788), which includes a genomic location with complementarity to the miR-483-5p (Figure 5A and 5B). These results indicate that endogenous Ago1 and Ago2 are associated with the P3 promoter and its adjacent downstream DNA fragment, rather than the distant downstream genomic fragment complementarity to miR-483-5p, and the association is miR-483-5p-dependent.

### Transcriptional up-regulation of P3 mRNA by miR-483-5p correlates with increased enrichment of RNA polymerase II and activating histone marks at the P3 promoter

To further explore that if the transcriptional up-regulation of P3 mRNA by miR-483-5p involves the changes of RNA polymerase II (RNAP II) occupancy and modification of histones at the P3 promoter, we performed ChIP assays using the same 5 primer pairs as the above covering the regions (Region A–E). The results showed that increased RNAP II occupancy in Region C and D, and increased enrichment of activating histone marks histone 3 lysine 4 trimethylation (H3K4me3), histone 3 lysine 27 acetylation (H3K27ac), and histone 4 lysine 5/8/12/16 acetylation (H4Kac) in Region A, B, C, and D were observed following treatment with miR-483-5p mimic, whereas treatment with miR-483-5p inhibitor decreased RNAP II occupancy and enrichment of the activating histone marks. Consistent with the above, in Region E, no significant RNAP II occupancy and enrichment of the activating histone marks was detected (Figure 5C–5F). Taken together, these results suggest that miR-483-5p-induced transcriptional up-regulation of P3 mRNA involves RNAP II enrichment and epigenetic changes associated with active transcription at the P3 promoter.

### Ago1 and Ago2 are required for P3 mRNA overexpression induced by miR-483-5p-mediated enrichment of RNAP II and activating histone marks at the P3 promoter

**Figure 6:**
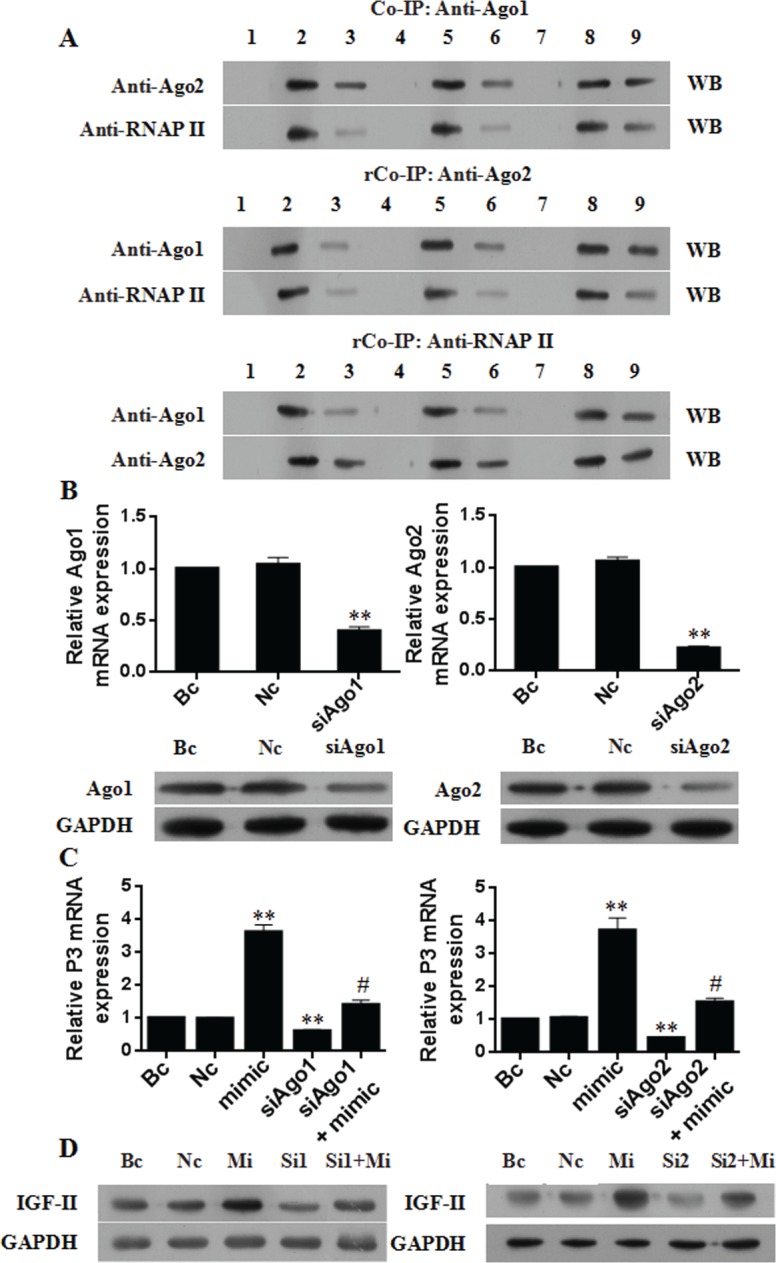
Ago1 and Ago2 are necessary for P3 mRNA transcription activation by miR-483-5p (**A**) Co-immunoprecipitation (Co-IP) assay and reversal co-immunoprecipitation (rCo-IP) assay results. Huh7 cells were transfected with 50 nM miR-483-5p mimic or scrambled control, and the nuclear extracts were used for Co-IP or rCo-IP. MiR-483-5p mimic treatment significantly increased the interactions of Ago1 and Ago2 with each other and with RNAP II, but it did affect the expression levels of Ago1, Ago2, and RNAP II proteins. IgG served as a negative control. (**B**) Effect of Ago1 or Ago2 knockdown by siRNA on the expression of Ago1 mRNA, Ago2 mRNA, Ago1 protein, and Ago2 protein. ^**^P < 0.01 versus Nc or Bc. (**C** and **D**) Effect of Ago1 or Ago2 knockdown by siRNA on the expression of P3 mRNA and IGF-II protein. Knockdown of Ago1 or Ago2 significantly decreased the induction of P3 mRNA (C) and IGF-II protein (D) by miR-483-5p, and the expression of endogenous P3 mRNA (C) and IGF-II protein (D) in the absence of ectopic miR-483-5p. ^**^P < 0.01 versus Nc or Bc. # < 0.01 versus miR-483-5p mimic. WB, Western blotting; Bc, blank control; Nc, negative control siRNA; Sc, scrambled control; mimic and Mi, miR-483-5p mimic; inhibitor, miR-483-5p inhibitor; Si1, siAgo1; Si2, siAgo2; 1 and 4 and 7, IgG; 2, Bc input; 5, Sc input; 8, Mi input; 3, Bc IP; 6, Sc IP; 9, Mi IP.

To further evaluate the necessity of Ago1 and Ago2 proteins for P3 mRNA overexpression by miR-483-5p, we first investigated whether Ago1 and Ago2 could directly interact with each other and with RNAP II. Huh7 cells were used for co-immunoprecipitation. The nuclear extracts were immunoprecipitated with anti Ago1 antibody, followed by Western blotting with anti-Ago2 or anti-RNAP II antibody. Reverse co-immunoprecipitation was performed with anti-Ago2 or anti-RNAP II antibody, followed by Western blotting with anti-Ago1, anti-Ago2 or anti-RNAP II antibody. The results showed that Ago1 and Ago2 directly interacted with each other and with RNAP II) (Figure 6A). Furthermore, miR-483-5p mimic treatment significantly increased the interactions between them (Figure 6A). These findings suggest that a multi-protein complex Ago1-Ago2-RNAP II may be formed *in vivo* and that miR-483-5p may induce their interactions.

Then, siRNAs were used to knock down the endogenous expression of Ago1 and Ago2, and the expression of P3 mRNA and IGF-II protein was examined in Huh7 cells. The siRNA specific for Ago1 or Ago2 resulted in about 60% Ago1 mRNA knockdown (Figure 6B) and 78% Ago2 mRNA knockdown (Figure 6B), respectively, and the knockdown of Ago1 or Ago2 protein was further confirmed by Western blotting (Figure 6B). Silencing of Ago1 or Ago2 caused a significant impairment of miR-483-5p-mediated activating effect on the expression of P3 mRNA (Figure 6C) and IGF-II protein (Figure 6D). Further results showed that Ago1 or Ago2 knockdown partially abolished enrichment of RNAP II and the above activating histone marks by miR-483-5p at the P3 promoter (Supplementary Figure 2A–2D). On the other hand, we transiently transfected Ago1 or Ago2 expression plasmid (pCMV3-Ago1 or pCMV3-Ago2) into Huh7 cells, respectively. The result showed the transient overexpression of Ago1 or Ago2 (Supplementary Figure 3A–3E) resulted in increased expression of P3 mRNA (Supplementary Figure 3F) and IGF-II protein (Supplementary Figure 3G and S3H), accompanied by increased enrichment of RNAP II (Supplementary Figure 4A) and the activating histone marks (Supplementary Figure 4B–4D) at the P3 promoter compared with negative control. Taken together, these data indicate both Ago1 and Ago2 are necessary for miR-483-5p-mediated P3 mRNA transcription activation.

### miR-483-5p promotes the proliferation of HCC cells

**Figure 7:**
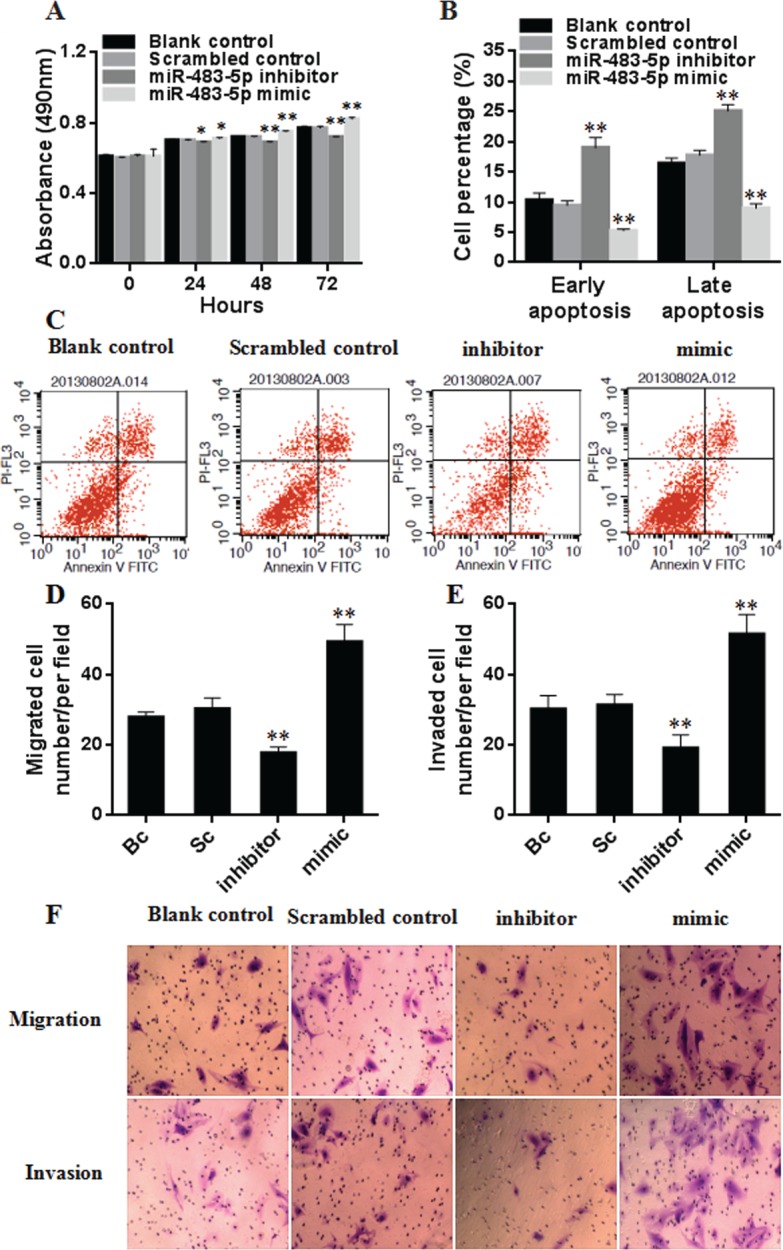
Effect of miR-483-5p on the proliferation, apoptosis, migration, and invasion of Huh7 cells (**A**) Cell proliferation was evaluated by MTT, and the results of cell growth were expressed by absorbance at 490 nm. ^*^P < 0.05, ^**^P < 0.01 versus scrambled control or blank control group. (**B** and **C**) Cell apoptosis was investigated by flow cytometry, and the results of early and late apoptotic cells were expressed by the percentage. ^**^P < 0.01 versus scrambled control or blank control group. (**D–F**) The capability of cell migration and invasion was evaluated by transwell assay, the results were expressed by the number of migrated and invaded cells. ^**^P < 0.01 versus scrambled control or blank control group. Bc, blank control; Sc, scrambled control; mimic, miR-483-5p mimic; inhibitor, miR-483-5p inhibitor.

To determine the effect of miR-483-5p on the proliferation of HCC cells, miR-483-5p mimic, miR-483 inhibitor, and scrambled control were transfected into Huh7 cells. MTT assay results showed that the ectopic overexpression of miR-483-5p promoted the proliferation of Huh7 cells (from 24 h to 72 h after transfection), and the suppression of the endogenous miR-483-5p expression reduced the proliferation of Huh-7 cells (from 24 h to 72 h after transfection) compared with scrambled control or blank control (Figure 7A). The findings suggest that miR-483-5p plays an important role in HCC cell proliferation.

### miR-483-5p inhibits the apoptosis of HCC cells

The effect of miR-483-5p on the apoptosis of HCC cells was investigated by flow cytometry. Compared with scrambled control or blank control, miR-483-5p mimic treatment resulted in a significant decrease in the apoptosis of Huh7 cells, while the cells treated with miR-483-5p inhibitor showed an inverse change in the apoptotic cells (Figure 7B and 7C). These results indicate that miR-483-5p suppresses the apoptosis of HCC cells.

### miR-483-5p promotes the migration and invasion of HCC cells

To test whether miR-483-5p enhances the capability of HCC cell migration and invasion, the transwell assays were performed. The migration test showed that miR-483-5p mimic treatment increased the migration rate of Huh7 cells, and miR-483 inhibitor treatment decreased their migration rate compared with scrambled control or blank control. Matrigel invasion test revealed that the number of cells that invaded to the lower chamber is significantly higher in the cells transfected with miR-483-5p mimic, and is significantly lower in the cells transfected with miR-483-5p inhibitor than that in the cells transfected with scrambled control or blank control (Figure 7D–7F). These results indicate that miR-483-5p enhances the invasion and migration capability of HCC cells.

### Correlation of miR-483-5p expression level with clinicopathological characteristics and prognosis of HCC patients

The clinicopathological characteristics of HCC patients are presented in Supplementary Table 2. The mean of the relative miR-483-5p expression level was 0.431 for all the HCC tissues studied. We arbitrarily considered samples with the relative miR-483-5p expression equal to or above 0.431 as high miR-483-5p expressers and samples with the relative miR-483-5p expression less than 0.431 were considered as low expressers. The HCC patients with high miR-483-5p expression were more often associated with high P3 mRNA expression, high AFP level, poor tumor differentiation (Edmondson grade III–IV), and tumor embolus of portal vein (TEPV) compared with the patients with low miR-483-5p expression, whereas there were no significant differences between the high and low miR-483-5p expression groups with respect to age, sex, tumor size, tumor nodularity, and HBV or HCV infection (Supplementary Table 2).

Kaplan–Meier method and the log-rank test showed that the HCC patients with high miR-483-5p expression had both shorter disease-free survival time and shorter overall survival time compared with the patients with low miR-483-5p expression (Supplementary Figure 5A and 5B). Univariate Cox regression analysis revealed that high AFP, poor tumor differentiation, TEPV, and high miR-483-5p expression were significantly associated with both shorter disease-free survival time and shorter overall survival time. Using multivariate Cox regression analysis, poor tumor differentiation and high miR-483-5p expression were found to be independent predictive factors for both shorter disease-free survival time and shorter overall survival time of the HCC patients (Supplementary Table 3).

## DISCUSSION

The overexpression of IGF-II is involved in the pathogenesis of a variety of human cancers, including cancers of the breast, bladder, prostate, colon, and liver [27–29]. In previous studies, we showed that increased IGF-II expression in HCC mainly resulted from the reactivation of the fetal P3 and P4 promoters [10, 18]. However, the mechanisms that may contribute to up-regulation of the two fetal transcripts (P3 mRNA and P4 mRNA) are not well understood.

In the present study, we showed that the 5′UTR of P3 mRNA only, rather than the other RNA transcripts and genomic sequece of the entire IGF-II gene, possessed a potential miR-483-5p-binding site by in silico analysis. qRT-PCR results indicated that the higher level of miR-483-5p was detected in the 5 human HCC cell lines and in the human HCC tissues compared with the human normal liver cell line-HL-7702 cells, and the MANT and the NALT, respectively. Linear correlation analysis revealed that P3 mRNA level was positively correlated to miR-483-5p level in the 5 human HCC cell lines, and in the human HCC tissues, respectively. These results suggest the possibility that miR-483-5p overexpression may play a significant role in the development of HCC and may be implicated in the positive regulation of P3 mRNA expression. Similar to our findings, several studies showed that the significant up-regulation of miR-483-5p was found in adrenocortical carcinoma [30–32] and pheochromocytoma [33].

To further investigate whether miR-483-5p induces P3 mRNA transcription through targeting the P3 mRNA 5′UTR, we performed a series of *in vitro* experiments. We first showed that miR-483-5p directly recognized the P3 mRNA 5′UTR to promote gene expression by luciferase assays in HeLa cells, 293T cells, and Huh7 cells. Then, the transient transfection experiments indicated that the treatment with miR-483-5p mimic or miR-483 inhibitor resulted in a significant increase or decrease in P3 mRNA expression in a dose-dependent manner in Huh7 and Hep3B cells, respectively. Consistent with the above results, Western blot analysis showed that miR-483-5p mimic or miR-483 inhibitor treatment increased or decreased IGF-II protein expression in the two cell lines, respectively. Finally, miR-483-5p expression study showed that mature miR-483-5p was present in both cytoplasm and nucleus of the HCC cell lines Hep3B and Huh7; the nuclear run-on assay revealed that miR-483-5p mimic or miR-483-5p inhibitor treatment induced or inhibited nascent P3 mRNA expression in the nucleus of Huh7 cells; the RNA stability assay indicated that miR-483-5p did not alter P3 mRNA stability in Huh7 cells. Collectively, the above results suggest that miR-483-5p induces P3 mRNA transcription in nucleus of the HCC cell lines by targeting the P3 mRNA 5′UTR.

It is widely accepted that most miRNAs target homologous sites in the 3′UTR of mRNAs to suppress translation or degrade mRNAs post-transcriptionally by interacting with Ago proteins in the cytoplasm [34, 35]. miRNAs also are able to target the 5′UTR [36] or coding region [37] of mRNAs for mediating translation repression. Furthermore, miRNAs are found to up-regulate translation by targeting 5′UTR [12] or 3′UTR [38] of mRNAs. It is noteworthy that a majority of miRNAs are imported back into the nucleus after maturation [20] with an abundance of putative miRNA target sites in gene promoters [25, 39], which suggests that miRNAs may also regulate gene expression at the transcriptional level. Indeed, it has been shown that miRNAs can up-regulate gene transcription by targeting gene promoters [26, 40, 41] or by targeting a non-coding promoter RNA [42], or down-regulate gene transcription by targeting a gene promoter [25] or by targeting a non-coding promoter RNA [43]. These findings raise the possibility of multiple modes and complexity of miRNA-mediated gene regulation.

In the current study, we further explored the possible mechanism involved in the transcriptional up-regulation of P3 mRNA by miR-483-5p. Firstly, we showed that the treatment with miR-483-5p mimic or miR-483-5p inhibitor significantly increased or decreased the specific recruitment of Ago1 and Ago2 to the 5′UTR of P3 mRNA, respectively, and that miR-483-5p could interact directly with Ago1 and Ago2 in the nucleus of Huh7 cells. These findings suggest that miR-483-5p binds to Ago1 and Ago2 and in turn recruits them to its complementary target site of the P3 mRNA 5′UTR in a sequence-specific manner. Next, we revealed that miR-483-5p mimic or miR-483-5p inhibitor treatment also significantly increased or decreased recruitment of Ago1 to the proximal P3 promoter region (−354/+459) and increased or decreased recruitment of Ago2 to the extended P3 promoter region (-841/+459) in Huh7 cells, respectively. On the other hand, increased or decreased RNAP II occupancy at the proximal P3 promoter region (-354/+459), and increased or decreased recruitment of activating histone marks H3K4me3, H3K27ac, and H4Kac to the extended P3 promoter region (-841/+459) were also detected following the treatment with miR-483-5p mimic or miR-483-5p inhibitor, respectively. These results suggest that miR-483-5p-mediated transcriptional up-regulation of P3 mRNA is involved in increased enrichment of Ago1 and Ago2, RNAP II, and activating histone marks at the P3 promoter. Then, co-immunoprecipitation analysis showed that Ago1 and Ago2 directly interact with each other and with RNAP II, and that the treatment with miR-483-5p mimic augmented the interaction between them *in vivo*, suggesting miR-483-5p may induce the formation of a multi-protein complex Ago1-Ago2-RNAP II in the nucleus of Huh7 cells. Finally, using siRNA knockdown assays, we found that Ago1 or Ago2 knockdown caused a significant decrease in the expression of P3 mRNA and IGF-II protein, accompanied by decreased enrichment of RNAP II and the above activating histone marks at the P3 promoter; contrarily, the transient overexpression of Ago1 or Ago2 induced the expression of P3 mRNA and IGF-II protein, accompanied by increased enrichment of RNAP II and the activating histone marks at the P3 promoter. These results suggest that Ago1 or Ago2 is required for miR-483-5p-mediated P3 mRNA transcription up-regulation, and Ago1 or Ago2 functions in the form of an intact multi-protein complex Ago1-Ago2.

Several studied have shown that miRNAs target gene promoters and up-regulate their transcriptions [26, 40, 41]. For example, let-7i recruits Ago1 and Ago2 to the IL-2 promoter by binding to the TATA-box motif of the promoter, and up-regulates its transcription activity in HEK293T cells [41]. On the other hand, miR-589 can target the non-coding RNA that overlap the COX-2 promoter and increases the binding of RNAi factors Ago2 and GW182 to the promoter RNA to activate COX-2 transcription by recruiting RNAP II to the COX-2 promoter. In this process, the promoter RNA acts as a scaffold for miR-589/RNAi factor complex [42]. Further, the report by Liu et al. showed that miR-483-5p could target the P3 mRNA 5′UTR of IGF-II gene rather than its promoter or promoter RNA, and in turn up-regulated its own transcription by enhancing the association of the RNA helicase DHX9 with the IGF2 transcript in MHH-ES-1 Ewing’s sarcoma cells [44]. This is a novel mechanism for miRNAs to up-regulate gene expression.

**Figure 8:**
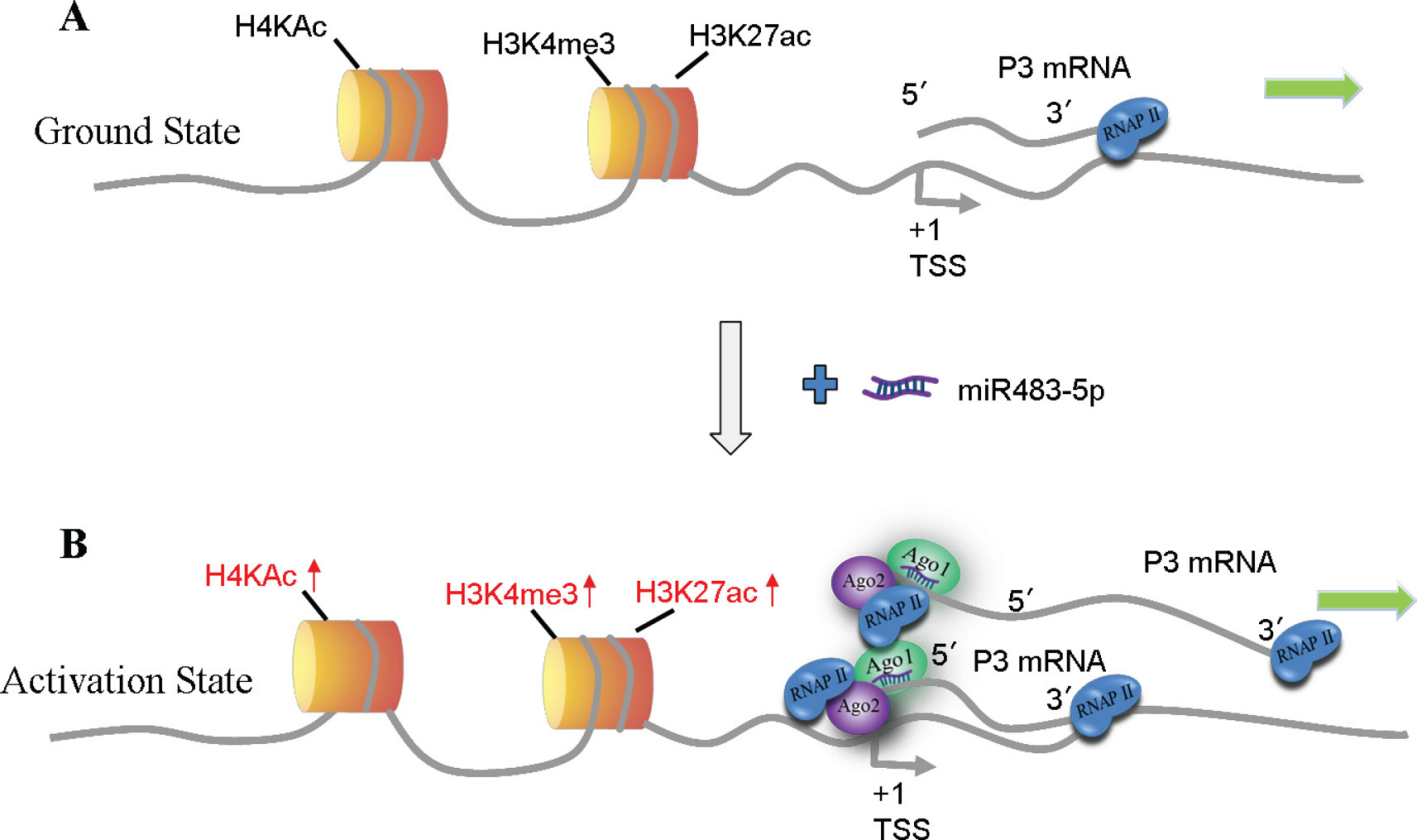
Model of miR-483-5p-mediated activation of P3 mRNA transcription (**A**) Ground state: P3 mRNA is expressed at low levels. (**B**) Gene activation state: binding of miR-483-5p/Ago1/ Ago2 complex to the complementary target site of the P3 mRNA 5′UTR in a sequence-specific manner, and the complex is also recruited to the P3 promoter due to spatial proximity, which further leads to enrichment of RNAP II and activating histone marks H3K4me3, H3K27ac, and H4Kac at the P3 promoter that activates P3 mRNA transcription. TSS, transcription start site.

In the present study, we identify another potential new model for miRNAs in up-regulating gene expression, in which miR-483-5p interacts with RNAi factors Ago1 and Ago2, and recruits them in a sequence-specific manner to its complementary target site of the P3 mRNA 5′UTR; in the mean time, RNAi factors Ago1 and Ago2 as an intact multi-protein complex are also recruited to the P3 promoter due to spatial proximity between the P3 mRNA 5′UTR and its promoter (Figure 8); further, RNAP II is enriched at the P3 promoter by the interaction with Ago1 and Ago2, accompanied by enrichment of activating histone marks H3K4me3, H3K27ac, and H4Kac at the P3 promoter, and then the transcription of P3 mRNA itself is activated. In the model, the P3 mRNA 5′UTR functions also as a scaffold for miR-483-5p/RNAi factors complex, which is similar to the COX-2 promoter RNA [42]. But the difference between them is very significant: the COX-2 promoter RNA acts only as a non-coding RNA, whereas P3 mRNA functions as both a non-coding RNA and a coding RNA. The differences between our study and the study by Liu et al. [44] are as follows. (1) We started this study in July 2012, and the *in vitro* function study with respect to miR-483-5p was completed in August 2013 (Figure 7B and 7C), whereas Liu et al. published their report in December 2013, suggesting that we and they performed the study associated with miR-483-5p independently, and that their study is earlier than ours. (2) In our study, miR-483-5p-mediated P3 mRNA transcription induction is involved in increased recruitment of RNAi factors Ago1 and Ago2 protein complex to its own 5′UTR and its own P3 promoter, which further results in increased enrichment of RNAP II and activating histone marks at the P3 promoter in the nucleus of Huh7 cells. In this process, the P3 mRNA 5′UTR itself functions also as a scaffold for miR-483-5p/RNAi factors complex. In the report by Liu et al. [44], miR-483-5p-mediated P3 mRNA transcription up-regulation is implicated in increased recruitment of DHX9 to the P3 mRNA 5′UTR in MHH-ES-1 Ewing’s sarcoma cells, but it remains unkown how DHX9 mediates P3 mRNA transcription activation.

Lines of evidence show that miRNAs are frequently deregulated in human cancer and contribute to the development and progression of cancer [45, 46]. miR-483-5p, a miRNA located in the intron 2 of IGF-II gene, is shown to be involved in the development of several cancers [30–32]. Soon et al. reported that high miR-483-5p expression predicted for poor overall specific survival in adrenocortical carcinomas [31]; Chabre et al. also found that high serum miR-483-5p level was associated with both shorter recurrence-free survival and shorter overall survival in adrenocortical cancer patients [32]; similarly, Wang et al. reported that high serum miR-483-5p concentration was related to poor survival outcome in patients with nasopharyngeal carcinoma [47]. On the other hand, Dong et al. reported that serum IGF-II level was correlated with extrahepatic metastases in HCC patients [48]; Xion et al. observed that the increase of IGF-II level in serum appeared to be associated with the occurrence of metastatic HCC after TACE and chemotherapy [49]; Sayer et al. found that high IGF-II expression was an independent predictor of poor survival in patients with epithelial ovarian cancer [50]. The above findings indicate that the overexpression of both miR-483-5p and IGF-II may be associated with poor clinical outcome in cancer.

In the present study, *in vitro* functional study showed that the ectopic overexpression of miR-483-5p led to increased proliferation, apoptosis inhibition, and increased migration and invasion capability in Huh7 cells, whereas the suppression of endogenous miR-483-5p expression showed an inverse change in the proliferation, apoptosis, migration and invasion capability in Huh7 cells. Clinicopathological data indicated that high miR-483-5p expression were significantly associated with high P3 mRNA expression, high AFP, poor tumor differentiation, and TEPV in patients with HCC. Kaplan–Meier analysis showed the correlation of high miR-483-5p expression with significantly poorer disease-free survival and overall survival; univariate and multivariate Cox regression analyses showed that high miR-483-5p expression could be one of independent predictive markers for shorter disease-free survival and overall survival time in the 83 patients who underwent curative resection for HCC. Moreover, it was found that high AFP, poor tumor differentiation, and TEPV were closely linked with shorter disease-free survival and overall survival time of HCC patients by univariate Cox regression analysis, and that poor tumor differentiation could be a significant independent prognostic marker for poor survival of HCC patients by multivariate Cox regression analysis.

Thus, high miR-483-5p expression is not only significantly correlated with more malignant tumor behaviors (increased migration and invasion capability, poor histological grade, portal vein invasion) that are important prognostic factors for HCC, but it is also correlated with poor survival of the HCC patients. Taken together, these findings suggest the possibility that miR-483-5p may play a significant role in the development and progression of HCC, and that high miR-483-5p expression may be related to an inferior clinical outcome for HCC patients.

In summary, the data presented in this study support an attractive and potentially novel model for miRNAs to up-regulating gene expression. In this model, miR-483-5p recruits RNAi factors complex Ago1-Ago2 in a sequence-specific manner to the complementary target site of the P3 mRNA 5′UTR, and the P3 promoter of its host gene, IGF-II gene, which further results in enrichment of RNAP II and the activating histone marks at the P3 promoter, and then the transcription of P3 mRNA itself is activated in the nucleus of HCC cells. In this process, the P3 mRNA5′UTR itself functions also as a scaffold for miR-483-5p/RNAi factors complex to activate its own transcription. High miR-483-5p expression may be involved in the development and progression of HCC at least in part through up-regulating P3 mRNA transcription, and may be associated with a poor prognosis of HCC patients. Our findings provide new information for understanding the mechanism for miRNAs to up-regulate gene expression in cancer.

## MATERIALS AND METHODS

### Cell lines and human tissue specimens

Huh7 (JCRB Cell Bank, Japan), Hep3B (ATCC, Manassas, USA), Bel-7402 (ATCC), HepG2 (ATCC), SMMC-7721 (ATCC), HL-7702 (ATCC), HeLa (ATCC), and 293T (ATCC) cell lines were cultured in Dulbecco’s modified Eagle medium (Gibco BRL, Rockville, USA) supplemented with heat-inactivated 10% fetal bovine serum (Gibco BRL). Tumor tissue specimens were obtained from 70 patients with HBV infection-positive/hepatitis C virus (HCV) infection-positive HCC (53 men, 17 women) and 13 patients with both HBV and HCV infection-negative HCC (11 men, 2 women) who underwent curative resection at the First Affiliated Hospital of Jinan University (Guangzhou, China) from January 2004 to December 2016. The diagnosis of HCC was made by a pathological examination, and all the patients did not receive any preoperative treatment before admission. The patients were followed up for 5-92 months. The patients with recurrence were submitted to standard treatments, such as re-resection, ablation or transarterial chemoembolization according to their condition. As control, normal adult liver tissue samples were obtained from 22 subjects (18 cases of hepatic hemangioma and 4 cases of hepatic rupture; 17 men, 5 woman). HBV infection was defined as positive detection of serum HBsAg and HBV DNA, and HCV infection was defined as positive detection of anti-HCV and HCV RNA. The study was approved by the medical ethics committee of the First Affiliated Hospital of Jinan University, and written informed consent was obtained from each participant.

### Quantitative RT-PCR (qRT-PCR)

Total RNA was extracted from the human HCC cell lines, HL-7702 cell line, and the human tissue specimens using Trizol reagent (Invitrogen, Carlsbad, CA, USA) according to the manufacturer’s protocol. For the expression analysis of miR-483-5p, qRT-PCR was carried out with TaqMan microRNA assay kits (Applied Biosystems, Foster City, CA, USA), and results were normalized to U6 RNA. For the expression analysis of P3 mRNA, Ago1 mRNA, and Ago2 mRNA, qRT-PCR was carried out using Power SYBR Green PCR Master Mix (Applied Biosystems), and results were normalized to GAPDH. Each experiment was repeated thrice and all reactions were carried out in triplicate. The qPCR primers used for miR-483-5p (MIMAT0004761), P3 mRNA (NM_000612), Ago1 mRNA (NM_012199.2), and Ago2 mRNA (NM_012154.2) are listed in Supplementary Table 4.

### Western blot analysis

Total protein from the human HCC cell lines was extracted using RIPA buffer (Pierce, Rockford, IL, USA). Equal amounts of protein for each sample were subjected to SDS-PAGE followed by transfer of protein to nitrocellulose membranes (Bio-Rad, Hercules, CA, USA). The membranes were first incubated with antibodies against IGF-II (Abcam, Cambridge, UK), Ago1 (Millipore, Billerica, MA, USA), Ago2 (Millipore), RNAP II (Millipore), α-tubulin (Abcam), Topoisomerase I (Abcam), and GAPDH (Abcam), then followed by incubation with HRP-conjugated secondary antibodies. GAPDH (Abcam) was served as a loading control. The results were visualized using an enhanced chemiluminescence detection system (Amersham Biosciences, Piscataway, NJ, USA). Bands were quantified with Image-Pro Plus software (Media Cybernetics, USA). Each experiment was repeated thrice and all reactions were carried out in triplicate.

### Nuclear run-on assay

Nuclear run-on assays were performed according to the method described by Zhang et al. with minor modifications [51]. Briefly, Huh7 cells were transfected with 50 nM miR-483-5p mimic, miR-483-5p inhibitor or scrambled control (Dharmacon, Lafayette, CO, USA) using Lipofectamine 2000 (Invitrogen). At 48 hours after transfection, the cells were harvested, and lysed on ice in cell lysis buffer [10 mM Tris-HCl (pH 7.4), 10 mM NaCl, 3 mM MgCl2, 100 mM sucrose, 0.5% NP-40]. The nuclei were collected and incubated in reaction buffer [5 mM Tris-HCl (pH 8.0), 2.5 mM MgCl2, 150 mM KCl, 2.5mM each of ATP, GTP, CTP and biotin-16-UTP] (Roche Molecular Biochemicals, Mannheim, Germany) for 45 min at 30°C. The reaction was stopped by the addition RNase-free DNase for 10 min at 37°C. Biotinylated nascent RNA transcripts were isolated by incubation with streptavidin Dynabeads^®^ (Invitrogen) and subjected to quantitative real-time RT-PCR for nascent P3 mRNA expression.

### mRNA stability assay

Huh7 cells were treated with miR-483-5p mimic or scrambled control (Dharmacon). Actinomycin D (Sigma-Aldrich, St.Louis, MO, USA) was used to block mRNA synthesis at 48 hours after treatment. The cells were collected at various time points and P3 mRNA level was quantified by qRT-PCR.

### Construction of luciferase reporter plasmids

The human full-length 5′UTR of P3 mRNA (NM_000612) containing the putative miR-483-5p binding sequences (216-238 nt) was cloned by RT-PCR from mRNAs isolated from Huh7 cells into the HindIII and NcoI restriction sites of pGL3 promoter vector (Promega, Madison, WI, USA). The recombinant luciferase reporter construct carrying the wild-type P3 mRNA 5′UTR was termed pGL3-P3-5′UTR-WT; the luciferase reporter construct carrying the mutated P3 mRNA 5′UTR sequence in the complementary sites for the seed region of miR-483-5p was generated based on pGL3-P3-5′UTR-WT by using a site-directed mutagenesis kit (Stratagene, La Jolla, CA, USA), termed as pGL3-P3-5′UTR-MUT. The mutant sequence was verified not to bind to known human miRNAs in silico using miRBase (Release 21.0, Sanger Institute: Cambridge, UK) and RegRNA programs. The primers used for the recombinant luciferase reporter construct are listed in Supplementary Table 4. All constructs were verified by sequencing.

### Cell transfection and dual-luciferase reporter assay

Cells were seeded in 24-well plates. After 24 h, each of these luciferase reporter constructs was transfected into the cells together with 50 nM miR-483-5p mimic, inhibitor or scrambled control (Dharmacon) and pRL-TK vector (Promega) for normalization of transfection efficiency using Lipofectamine 2000 (Invitrogen). At 48 h after transfection, the luciferase activities were measured using the Dual-Luciferase Report Assay (Promega).

### RNA immunoprecipitation /Chromatin immunoprecipitation

RNA immunoprecipitation (RIP) /chromatin immunoprecipitation (ChIP) assays were performed as described by Matsui et al. [52]. Briefly, Huh7 cells were transfected with 50 nM miR-483-5p mimic, miR-483-5p inhibitor or scrambled control (Dharmacon). At 72 hours after transfection, the cells were crosslinked with 1% formaldehyde. The cells were harvested and nuclei were isolated using hypotonic lysis buffer (10 mM Tris-HCl (pH 7.5), 10 mM NaCl, 3 mM MgCl2, 0.5% NP-40). Nuclei were lysed in lysis buffer [1% SDS, 10 mM EDTA, 50 mM Tris-HCl (pH 8.1), 1×Roche protease inhibitors cocktail, RNasin Plus RNase inhibitor (Promega)] and sonicated.

The nuclear extract was incubated overnight with antibodies in immunoprecipitation buffer. After antibody-protein-DNA/RNA complex was recovered with protein G plus/protein A agarose beads (Calbiochem, Darmstadt, Germany), the beads were washed with buffers. The complex was eluted twice with elution buffer. Crosslinking was reversed by adding NaCl to 200 mM and heating at 65°C for at least 2 hours. Protein was digested by incubating with proteinase K (Invitrogen) at 42°C for 50 min, followed by phenol-chloroform extraction and ethanol precipitation. For RIP, the samples were resuspended in nuclease-free water and treated with DNase I at 25°C for 10 min. Reverse transcription (RT) reactions were performed, then the samples were analyzed by qPCR. For ChIP, the samples were resuspended in nuclease-free water and analyzed by qPCR. For ChIP–immunoblot analysis, Huh7 cells were transfected with 50 nM biotin-labeled miR-483-5p mimic or scrambled control (Dharmacon) for 72 hours. Immunoprecipitation was performed with Dynabeads M-280 Streptavidin (Invitrogen) followed by immunoblotting with anti-Ago1 or anti-Ago2 antibody.

Antibodies used in RIP/ChIP were as follows: anti-RNAP II (Millipore), anti-H3K4me3 (Abcam), anti-H3K27ac (Millipore), anti-H4KAc (Millipore), anti-Ago1 (Millipore), anti-Ago2 (Millipore). Normal mouse immunoglobulin G (IgG) (Millipore) was used as a negative control. The sequences of qPCR primers used in RIP or ChIP are listed in Supplementary Table 4.

### Co-immunoprecipitation

Co-immunoprecipitation assays were performed by protein A and protein G conjugated magnetic beads (Invitrogen). 500 μg of nuclear extract of Huh7 cells was immunoprecipitated with anti-Ago1 (Millipore) or anti-Ago2 (Millipore), anti-RNAP II (Millipore) antibodies overnight at 4°C. Protein binding was detected by Western blotting with the respective antibodies.

### Small interfering RNA (siRNA) knockdown

Huh7 cells were transfected with siRNA oligonucleotides by using Lipofectamine RNAiMAX (Invitrogen). The sequences of the siRNAs used in the present study for Ago1 siRNA (siAgo1) (NM_012199.2) and Ago2 siRNA (siAgo2) (NM_012154.2) are listed in Supplementary Table 4. Negative control siRNA was purchased from Dharmacon. The cells were harvested at 72 h after transfection, and then the endogenous Ago1 and Ago2 mRNA and protein levels were determined.

### MTT assay

Huh7 cells were seeded into 96-well culture plates at a density of 1 × 104 cells per well, treated with miR-483-5p mimic, miR-483-5p inhibitor or scrambled control (Dharmacon). At the indicated time points (0, 24, 48 and 72 h after culture), 20 μL of 5 mg/mL MTT (Sigma, St. Louis, MO, USA) were added to each well, and the plates were incubated at 37°C for an additional 4 h. Then, the cells were lysed in dimethyl sulfoxide, and the optical density at 490 nm was determined. Each point was determined in triplicate and an average was obtained for analysis. The experiment was independently repeated three times.

### Cell apoptosis detection

Following treatment for 48 h as described above, Huh7 cells were harvested, washed with PBS, resuspended in the binding buffer, and incubated for 15 min in the dark with propidium iodide (PI) and Annexin V/fluorescein isothiocyanate (FITC) (Beyotime Institute of Biotechnology, Jiangsu, China). Apoptosis was then detected by a flow cytometer (Beckman Coulter, Brea, CA, USA). The experiment was independently repeated three times.

### Transwell cell migration/invasion assays

The Transwells (Costar, Cambridge, MA, USA) with 8 μm pore polycarbonate membranes (Corning Inc., Corning, NY, USA) were left uncoated or were coated with Matrigel (BD Biosciences, Franklin Lakes, NJ, USA) before use in migration assays and invasion assays, respectively. Matrigel was added into each Transwell upper chamber and placed in a 37°C incubator for 2 h to solidify. Following treatment for 48 h as described above, Huh7 cells were seeded in the transwell upper chamber. The transwell assays were performed according to the manufacturer’s instructions. After transwell chambers were incubated for 24 hours at 37°C in a humidified incubator with 5% CO_2_, the lower chamber was stained with crystal violet. Cell migration/invasion was evaluated by counting the cells that had migrated /invaded into the filters. Each assay was performed in triplicate and repeated three times.

### Statistical analysis

Categorical data were evaluated by χ2 or Fisher’s exact tests, depending on the absolute numbers included in the analysis, quantitative data were analyzed by independent sample *t* test followed by Mann-Whitney *U* test, and linear correlation was evaluated by Pearson correlation coefficient. The Kaplan–Meier method and logrank test were used for survival analysis. Overall survival time was defined as the duration from the date of resection to the date of death from HCC. Disease-free survival time was defined as the duration from the date of resection to the date of tumor recurrence or metastasis. Independent prognostic factors were evaluated by Cox regression analysis. SPSS 18.0 software (SPSS Inc., Chicago, IL, USA) was used for all statistical analysis. Results were considered statistically significant at *P* < 0.05.

## SUPPLEMENTARY MATERIALS FIGURES AND TABLES





## Abbreviations

IGF-II: insulin-like growth factor-II
5′UTR: 5′-untranslated region
RIP: RNA immunoprecipitation
ChIP: chromatin immunoprecipitation
qRT-PCR: quantitative RT-PCR
siRNA: small interfering RNA
Ago: argonaute
RNAP II: RNA polymerase II
