# Supplementary material for: MiR-483-5p promotes IGF-II transcription and is associated with poor prognosis of hepatocellular carcinoma
Source: Oncotarget. 2017 Oct 11;8(59):99871–88. doi: 10.18632/oncotarget.21737 (PMC5725137; doi:10.18632/oncotarget.21737)
Supplement: Supplementary file 1 [file oncotarget-08-99871-s001.pdf]

MiR-483-5p promotes IGF-II transcription and is associated with poor prognosis of hepatocellular carcinoma

SUPPLEMENTARY MATERIALS

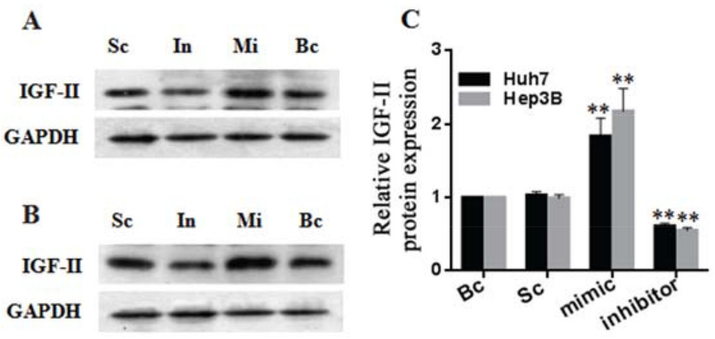

Supplementary Figure 1: Effect of miR-483-5p on IGF-II protein expression in Huh7 and Hep3B cells. (**A–C**) miR-483-5p mimic or inhibitor promoted or inhibited IGF-II protein expression in Huh7 (A, C) and Hep3B (B, C) cells, respectively. $^{**}P < 0.01$ versus scrambled or blank control. Bc, blank control; Sc, scrambled control; mimic and Mi, miR-483-5p mimic; inhibitor and In, miR-483-5p inhibitor.

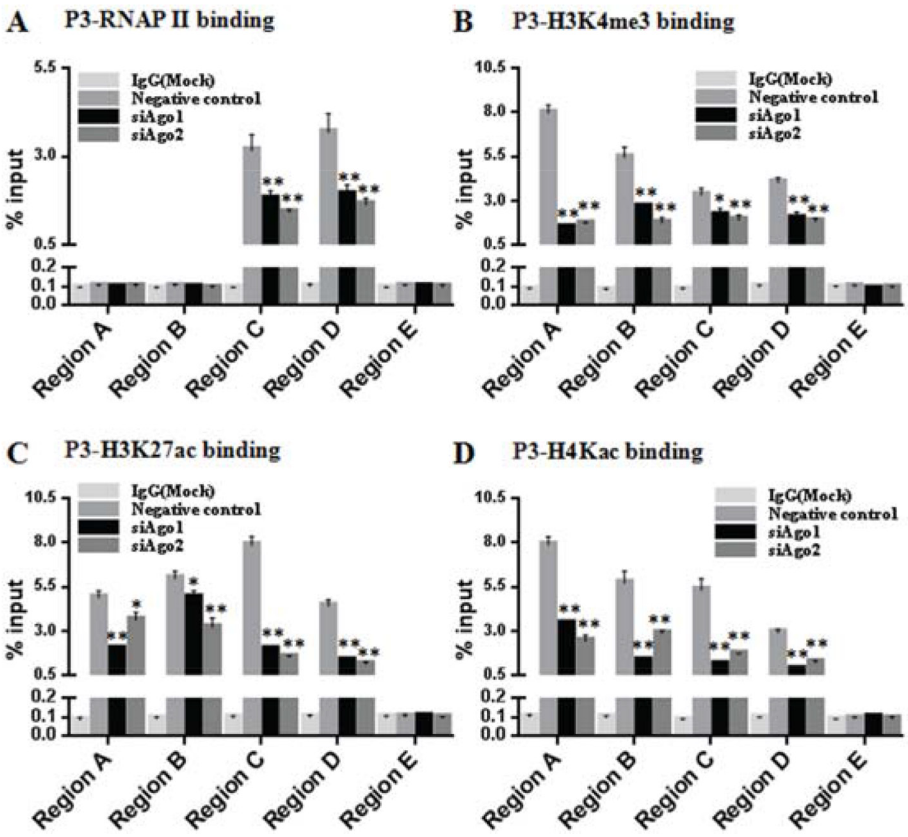

Supplementary Figure 2: Effect of Ago1 or Ago2 knockdown by siRNA on enrichment of RNAP II and activating histone marks at the P3 promoter in Huh7 cells. (**A–D**) Depletion of Ago1 or Ago2 by siRNA resulted in the significant decrease in enrichment of RNAP II (A) and activating histone marks H3K4me3 (B), H3K27ac (C), and H4Kac (D) compared with negative control. $^{*}P < 0.05$, $^{**}P < 0.01$ versus negative control. P3, P3 promoter; negative control, negative control siRNA.

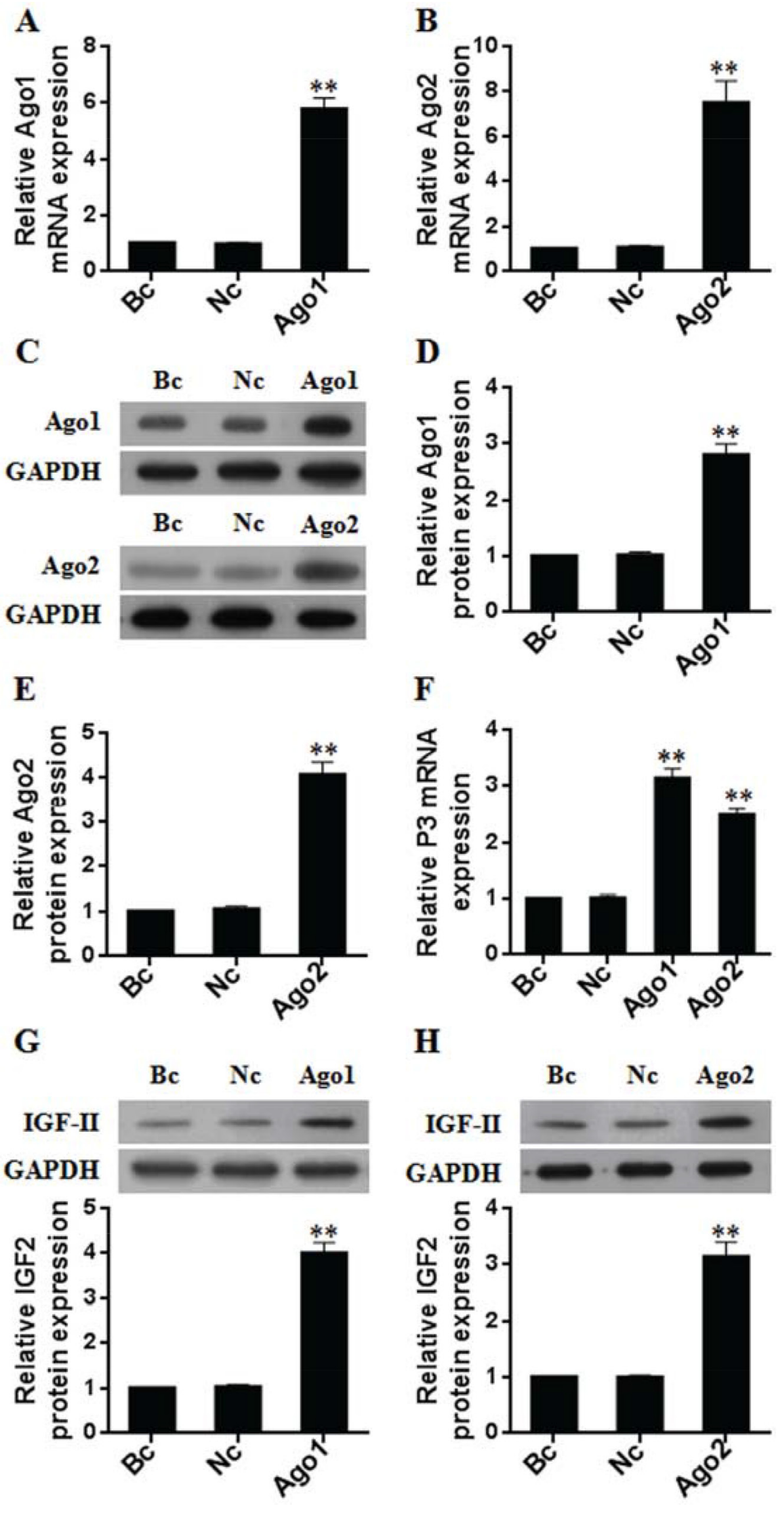

Supplementary Figure 3: Effect of the transient overexpression of Ago1 or Ago2 on the expression of P3 mRNA and IGF-II protein in Huh7 cells. Human Ago1 or Ago2 expression plasmid pCMV3-Ago1 or pCMV3-Ago2 (Sino Biological Inc., Beijing, China) was transfected into Huh7 cells, and the transfected quantities of pCMV3-Ago1 or pCMV3-Ago2 plasmid were 1.0 µg per 1×106 cells. (**A–E**) The transient transfection of pCMV3-Ago1 or pCMV3-Ago2 resulted in increased expression of Ago1 mRNA (A) or Ago2 mRNA (B), and Ago1 protein (C, D) or Ago2 protein (C, E), respectively. $^{**}P < 0.01$ versus negative or blank control. (**F–H**) The transient overexpression of Ago1 or Ago2 significantly increased the expression of P3 mRNA (F) and IGF-II protein (G, H), respectively. $^{**}P < 0.01$ versus negative or blank control. Bc, blank control; Nc, negative control.

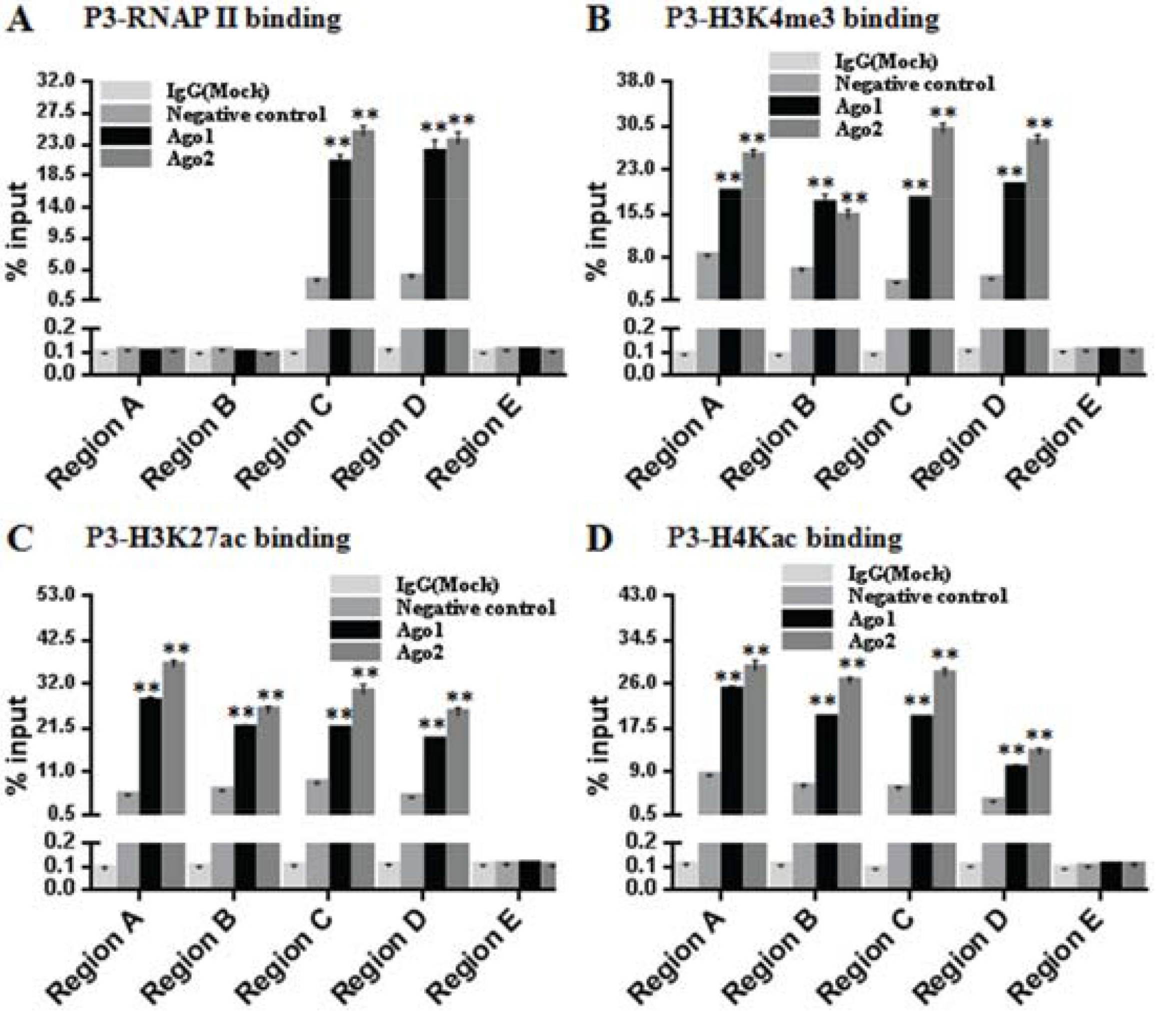

Supplementary Figure 4: Effect of Ago1 or Ago2 transient overexpression on enrichment of RNAP II and activating histone marks at the P3 promoter in Huh7 cells. (**A–D**) The transient overexpression of Ago1 or Ago2 significantly increased enrichment of RNAP II (A) and activating histone marks H3K4me3 (B), H3K27ac (C), and H4Kac (D) compared with negative control. $^{**}P < 0.01$ versus negative control. P3, P3 promoter.

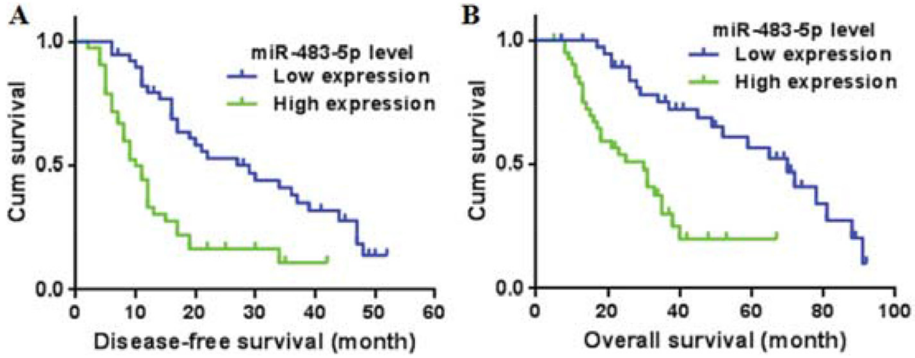

Supplementary Figure 5: Kaplan–Meier curves for disease-free survival and overall survival time in 40 patients with miR-483-5p low level and 43 patients with miR-483-5p high level. (**A**) The patients with miR-483-5p high level had a shorter disease-free survival time than those with miR-483-5p low level. (**B**) The patients with miR-483-5p high level had a shorter overall survival time than those with miR-483-5p low level.

Supplementary Table 1: The possible miRNAs binding to the P3 mRNA 5′UTR of IGF-II gene only. See Supplementary Table 1

Supplementary Table 2: Comparisons of clinicopathological data and experimental results between high and low miR-483-5p expressers

Parameters	miR-483-5pmiR-483-5p*P*		
	(high, *n* = 43)	(low, *n* = 40)	
Mean age (years)	54.49 ± 12.90	52.15 ± 11.50	0.869
Mean tumor diameter (cm)	8.59 ± 3.61	7.40 ± 2.50	0.086
Relative P3 mRNA level			
(mean [SD])	6.79 ± 6.18	0.91 ± 1.26	0.000
(median [range])	4.14 (0.03–23.95)	0.335 (0.003–5.35)	0.000
No. of female patients	8	11	0.335
No. of patients with AFP ≥ 400 μg/L	31	17	0.006
No. of patients with Multinodular	8	10	0.480
No. of patients with Edmondson grade III–IV	35	15	0.000
No. of patients with TEPV	35	12	0.000
No. of patients with HBV or HCV infection	39	31	0.098

Abbreviations: BNC-HCC, HBV infection-positive/ HCV infection-negative HCC; NBNC-HCC, Both HBV and HCV infection-negative HCC; TEPV, Tumor embolus of portal vein; AFP, α-Fetoprotein.

Supplementary Table 3: Univariate and multivariate Coxregression analysis showing disease-free survival and overall survival in 83 patients with HCC

Parameters	Disease-free survival			Overall survival		
	RR	95% CI	*P* value	RR	95% CI	*P* value
**Univariate**						
Gender	0.773	0.431–1.389	0.390	0.701	0.369–1.332	0.278
M: *n* = 64						
F: *n* = 19						
Age (years)	1.014	0.606–1.697	0.958	0.848	0.464–1.548	0.590
< 60: *n* = 52						
≥ 60: *n* = 31						
Tumor diameter (cm)	1.036	0.944–1.137	0.451	1.040	0.936–1.155	0.467
AFP (μg/L)	2.034	1.204–3.434	0.008	2.674	1.412–5.065	0.003
< 400: *n* = 35						
≥ 400: *n* = 48						
Tumor differentiation (Edmondson grade)	0.135	0.070–0.259	0.000	0.146	0.072–0.297	0.000
Well (I–II): *n* = 33						
Poor (III–IV): *n* = 50						
TEPV	2.321	1.379–3.907	0.002	3.241	1.746–6.015	0.000
Absence: *n* = 36						
Presence: *n* = 47						
Hepatitis infection (HBV or HCV)	1.782	0.908–3.497	0.093	2.031	0.905–4.561	0.086
Positive: *n* = 70						
Negative: *n* = 13						
Relative miR-483-5p level	2.886	1.684–4.947	0.000	3.765	1.932–7.340	0.000
High: *n* = 43						
Low: *n* = 40						
**Multivariate**						
Tumor differentiation (Edmondson grade)	0.156	0.080–0.308	0.000	0.182	0.086–0.384	0.000
Well (I–II): *n* = 33						
Poor (III–IV): *n* = 50						
Relative miR-483-5p1 level	889	1.070–3.334	0.028	2.157	1.073–4.337	0.031
High: *n* = 43						
Low: *n* = 40						

Abbreviations: M, Male; F, Female; AFP, α-Fetoprotein; TEPV, Tumor embolus of portal vein; CI, confidence interval.

Supplementary Table 4: DNA or RNA oligonucleotides

qPCR primers	Forward primer (5′–3′)	Reverse primer (5′–3′)
miR-483-5p	ACACTCCAGCTGGGAAGACGGG AGGAA AGA A	CTCAACTGGTGTCGTGGA
P3 mRNA	CTTTCACGTTCACTCTGTCTCT	GCCAGATGTTGTACTTTTCG
Ago1mRNA	TCTCAGCCACTGCCTTTTAT	ATCTCCTTGGTGAAGCGAAC
Ago2 mRNA	AGAAGTGCCCGAGGAGAGTT	TGATGGACACCTTGAAGATGC
**PCR primers for RLRC**	**Forward primer (5′–3′)**	**Reverse primer (5′–3′)**
pGL3-P3-5′UTR-WT	CCCAAGCTTCGCCTGTCCCCCTCCCGAGG	CATGCCATGGTGGTGTCTGGAAG CCGGCGACGC
pGL3-P3-5′UTR-MUT	CCGCAACCTTCCCTTCGCTCTTGTTT ACTCCCCCCAGCTCCTAGCCTCC	GGAGGCTAGGAGCTGGGGGGAGT AAACAAGAGCGAAGGGAAGGTT GCGG
**siRNA**	**Sense (5′–3′)**	**Antisense (5′–3′)**
siAgo1	GCUGGACAUCAGGAACAU ATT	UAUGUUCCUGAUGUCCAGCTT
siAgo2	GAC GGC AGG AAG AAU CUA UTT	AUA GAU UCU UCC UGC CGU CTT
**RIP primer**	**Forward primer (5′–3′)**	**Reverse primer (5′–3′)**
	GCAACCTTCCCTTCGCT	CACACGCCGCTTACCTG
**ChIP primers**	**Forward primer (5′–3′)**	**Reverse primer (5′–3′)**
Region A	CAACGCACTGAGGACGG	TGCCCCAGCAGAAGGAG
Region B	GTTTCCCGCCCTGATCCTC	CCATGCTGAATGCCCGTTCTA
Region C	CCAGCTCACGAGCACAGG	GCCTTGCCCGATGGAG
Region D	GCCGCCCGCTGCC	GCCCTCTGCCGTCGC
Region E	CCGCTCCCCTTTCCG	GAGAGGCGGGCAGGC

RLRC: the recombinantluciferase reporter construct.
