# Supplementary material for: MiR-483-5p promotes IGF-II transcription and is associated with poor prognosis of hepatocellular carcinoma
Source: Oncotarget. 2017 Oct 11;8(59):99871–88. doi: 10.18632/oncotarget.21737 (PMC5725137; doi:10.18632/oncotarget.21737)
Supplement: Supplementary file 2 [file oncotarget-08-99871-s002.docx]

**Supplementary Table 1: The possible miRNAs binding to the P3 mRNA 5′UTR of IGF-II gene only**

| **miRNAs** | **Location** | **Length**  **(bp)** | **Hybridization** | **Minimum Free Energy** |
| --- | --- | --- | --- | --- |
|  |  |  |  |  |
|  |  |  |  |  |
|  |  |  |  |  |
| hsa-miR-1915* | [19~43](http://regrna.mbc.nctu.edu.tw/php/showtable.php?ColorRegion=19~43&FileDir=tmp/20161027/085354&SeqID=085354&MotifInfo=miRNA%20Target%20Sites&MotifType=miranda) | 25 | miRNA: 3' ccGGGCCCG-UCGUU-CCGU-UCCa 5' | -33.2 |
|  |  |  | \|\|\|\|\|\|\| \|\|:\| \|\|\|\| \|\|\| |  |
|  |  |  | Target:5' ggCCCGGGCTCGCGACGGCAGAGGg 3' |  |
|  |  |  |  |  |
| hsa-miR-658 | [127~152](http://regrna.mbc.nctu.edu.tw/php/showtable.php?ColorRegion=127~152&FileDir=tmp/20161027/085354&SeqID=085354&MotifInfo=miRNA%20Target%20Sites&MotifType=miranda) | 26 | miRNA: 3' ugGUUGC-CUGGAUGAAGGGAGGCGg 5' | -33 |
|  |  |  | \|:\|\|\| \|:\| \|\|\|\|\|\|\|\|\|\|\| |  |
|  |  |  | Target:5' ctCGACGTGGCGCCCTTCCCTCCGCt 3' |  |
|  |  |  |  |  |
| hsa-miR-1268 | [263~280](http://regrna.mbc.nctu.edu.tw/php/showtable.php?ColorRegion=263~280&FileDir=tmp/20161027/085354&SeqID=085354&MotifInfo=miRNA%20Target%20Sites&MotifType=miranda) | 18 | miRNA: 3' ggGGGUGGUGGUGCGGGc 5' | -32.8 |
|  |  |  | \|\|\| \|\| :\|\|\|\|\|\|\| |  |
|  |  |  | Target:5' ctCCCCCCCTCACGCCCg 3' |  |
|  |  |  |  |  |
| hsa-miR-483-5p | [216~238](http://regrna.mbc.nctu.edu.tw/php/showtable.php?ColorRegion=216~238&FileDir=tmp/20161027/085354&SeqID=085354&MotifInfo=miRNA%20Target%20Sites&MotifType=miranda) | 23 | miRNA: 3' gaGGGAAG-AAAGGAGGGCAGaa 5' | -30.2 |
|  |  |  | \|\|\|\|\|\| \| \|\|\|\|\|\|\|\|\| |  |
|  |  |  | Target:5' ttCCCTTCGCTCCCTCCCGTCcc 3' |  |
|  |  |  |  |  |
| hsa-miR-1182 | [3~25](http://regrna.mbc.nctu.edu.tw/php/showtable.php?ColorRegion=3~25&FileDir=tmp/20161027/085354&SeqID=085354&MotifInfo=miRNA%20Target%20Sites&MotifType=miranda) | 23 | miRNA: 3' caguguaGGGAGGGUUCUGGGag 5' | -29.5 |
|  |  |  | \|\|\|\|\|\|\|:\|\|:\|\|\| |  |
|  |  |  | Target:5' cctgtccCCCTCCCGAGGCCCgg 3' |  |
|  |  |  |  |  |
| hsa-miR-3656 | [266~282](http://regrna.mbc.nctu.edu.tw/php/showtable.php?ColorRegion=266~282&FileDir=tmp/20161027/085354&SeqID=085354&MotifInfo=miRNA%20Target%20Sites&MotifType=miranda) | 17 | miRNA: 3' gguGGGGGCGUGGGCGg 5' | -29.3 |
|  |  |  | \|\|\|:\| \|:\|\|\|\|\| |  |
|  |  |  | Target:5' cccCCCTCACGCCCGCc 3' |  |
|  |  |  |  |  |
| hsa-miR-3180-3p | [87~104](http://regrna.mbc.nctu.edu.tw/php/showtable.php?ColorRegion=87~104&FileDir=tmp/20161027/085354&SeqID=085354&MotifInfo=miRNA%20Target%20Sites&MotifType=miranda) | 18 | miRNA: 3' ccGGAGGCCUUCGAGGCGGGGu 5' | -29 |
|  |  |  | \|\|\|\|\| \| \|\|\|\|\|\|\|\|\| |  |
|  |  |  | Target:5' agCCTCC---A-CTCCGCCCCc 3' |  |
|  |  |  |  |  |
| hsa-miR-3677 | [13~34](http://regrna.mbc.nctu.edu.tw/php/showtable.php?ColorRegion=13~34&FileDir=tmp/20161027/085354&SeqID=085354&MotifInfo=miRNA%20Target%20Sites&MotifType=miranda) | 22 | miRNA: 3' ccGGCACCGGUCUCGGGUGCUc 5' | -28.7 |
|  |  |  | \|\|\| \|\|\|\| \|:\|\|:\|:\|\|\| |  |
|  |  |  | Target:5' tcCCGAGGCCCGGGCTCGCGAc 3' |  |
|  |  |  |  |  |
| hsa-miR-615-3p | [11~32](http://regrna.mbc.nctu.edu.tw/php/showtable.php?ColorRegion=11~32&FileDir=tmp/20161027/085354&SeqID=085354&MotifInfo=miRNA%20Target%20Sites&MotifType=miranda) | 22 | miRNA: 3' uucuccCUCUGGGUCCGAGCcu 5' | -28.6 |
|  |  |  | \|\|\|:\|\|\|:\|\|\|\|\|\| |  |
|  |  |  | Target:5' cctcccGAGGCCCGGGCTCGcg 3' |  |
|  |  |  |  |  |
| hsa-miR-1224-5p | [365~384](http://regrna.mbc.nctu.edu.tw/php/showtable.php?ColorRegion=365~384&FileDir=tmp/20161027/085354&SeqID=085354&MotifInfo=miRNA%20Target%20Sites&MotifType=miranda) | 20 | miRNA: 3' ggUGGAGGGC-UCAGGAGug 5' | -28.1 |
|  |  |  | :\|\|\|\|:\|\| \|\|\|\|\|\| |  |
|  |  |  | Target:5' ccGCCTCTCGCTGTCCTCtc 3' |  |
|  |  |  |  |  |
| hsa-miR-1976 | [654~679](http://regrna.mbc.nctu.edu.tw/php/showtable.php?ColorRegion=654~679&FileDir=tmp/20161027/085354&SeqID=085354&MotifInfo=miRNA%20Target%20Sites&MotifType=miranda) | 29 | miRNA: 3' ugUCGU----UCCUC-----CCGUCCUCc 5' | -26.6 |
|  |  |  | :\|\|\| \|\|\|\|\| \|\|\|\|\|\|\|\| |  |
|  |  |  | Target:5' ctGGCAGXXXAGGAGTGTCCGGCAGGAGg 3' |  |
|  |  |  |  |  |
| hsa-miR-4270 | [213~232](http://regrna.mbc.nctu.edu.tw/php/showtable.php?ColorRegion=213~232&FileDir=tmp/20161027/085354&SeqID=085354&MotifInfo=miRNA%20Target%20Sites&MotifType=miranda) | 20 | miRNA: 3' cgGGAGGGGACUGAGGGAcu 5' | -26.4 |
|  |  |  | \|:\|\|\|\|:\| :\|\|\|\|\|\| |  |
|  |  |  | Target:5' acCTTCCCTTCGCTCCCTcc 3' |  |
|  |  |  |  |  |
| hsa-miR-3130-3p | [70~89](http://regrna.mbc.nctu.edu.tw/php/showtable.php?ColorRegion=70~89&FileDir=tmp/20161027/085354&SeqID=085354&MotifInfo=miRNA%20Target%20Sites&MotifType=miranda) | 20 | miRNA: 3' aauGGGUCAGAGGCCACGUCg 5' | -25.9 |
|  |  |  | \| \|:\|\|\| \| \|\|\|\|\|\|\| |  |
|  |  |  | Target:5' gccCGCGGTC-CGGGTGCAGc 3' |  |
|  |  |  |  |  |
| hsa-miR-328 | [660~684](http://regrna.mbc.nctu.edu.tw/php/showtable.php?ColorRegion=660~684&FileDir=tmp/20161027/085354&SeqID=085354&MotifInfo=miRNA%20Target%20Sites&MotifType=miranda) | 25 | miRNA: 3' ugCCUU-C---CCGUCUCUCCCGGUc 5' | -25.9 |
|  |  |  | \|\|\|: \| \|\|\|\|\| \|\|\|\|\|\|\|\| |  |
|  |  |  | Target:5' xaGGAGTGTCCGGCAG-GAGGGCCAa 3' |  |
|  |  |  |  |  |
| hsa-miR-3648 | [62~79](http://regrna.mbc.nctu.edu.tw/php/showtable.php?ColorRegion=62~79&FileDir=tmp/20161027/085354&SeqID=085354&MotifInfo=miRNA%20Target%20Sites&MotifType=miranda) | 18 | miRNA: 3' ggGAGCCGCUAGGGGCGCCGa 5' | -25.6 |
|  |  |  | \|\| \|\|\|\| \|\|\|\|\|\|\|: |  |
|  |  |  | Target:5' agCTGGGCG---CCCGCGGTc 3' |  |
|  |  |  |  |  |
| hsa-miR-3132 | [423~451](http://regrna.mbc.nctu.edu.tw/php/showtable.php?ColorRegion=423~451&FileDir=tmp/20161027/085354&SeqID=085354&MotifInfo=miRNA%20Target%20Sites&MotifType=miranda) | 29 | miRNA: 3' agGAGAC-UCGAG---GA-AGAGAUGGGu 5' | -25.3 |
|  |  |  | \|\|\|\|\| \|\|\| \|\| \|\|\|\|\|:\|\|\| |  |
|  |  |  | Target:5' caCTCTGTCTCTCCCACTATCTCTGCCCc 3' |  |
|  |  |  |  |  |
| hsa-miR-3162 | [211~232](http://regrna.mbc.nctu.edu.tw/php/showtable.php?ColorRegion=211~232&FileDir=tmp/20161027/085354&SeqID=085354&MotifInfo=miRNA%20Target%20Sites&MotifType=miranda) | 22 | miRNA: 3' gagGGGUGGGAAGAUGAGGGAuu 5' | -24.8 |
|  |  |  | \|\|: \|\|\|\|\|\| :\|\|\|\|\|\| |  |
|  |  |  | Target:5' caaCCTTCCCTTC-GCTCCCTcc 3' |  |
|  |  |  |  |  |
| hsa-miR-3944 | [510~538](http://regrna.mbc.nctu.edu.tw/php/showtable.php?ColorRegion=510~538&FileDir=tmp/20161027/085354&SeqID=085354&MotifInfo=miRNA%20Target%20Sites&MotifType=miranda) | 29 | miRNA: 3' ggccUCGUCGUCC------GGUCGGGCUu 5' | -24.4 |
|  |  |  | \| \|\| \| \|\| \|\|\|\|\|\|\|\|\| |  |
|  |  |  | Target:5' gtacAACATCTGGCCCGCCCCAGCCCGAa 3' |  |
|  |  |  |  |  |
| hsa-miR-3154 | [201~225](http://regrna.mbc.nctu.edu.tw/php/showtable.php?ColorRegion=201~225&FileDir=tmp/20161027/085354&SeqID=085354&MotifInfo=miRNA%20Target%20Sites&MotifType=miranda) | 25 | miRNA: 3' agACGAGG--GUU-GAGGGGAAGac 5' | -24.2 |
|  |  |  | \| \|\|\|\| \|\|\| \|\|:\|\|\|\|\|\| |  |
|  |  |  | Target:5' ctTTCTCCCGCAACCTTCCCTTCgc 3' |  |
|  |  |  |  |  |
| hsa-miR-501-3p | [68~89](http://regrna.mbc.nctu.edu.tw/php/showtable.php?ColorRegion=68~89&FileDir=tmp/20161027/085354&SeqID=085354&MotifInfo=miRNA%20Target%20Sites&MotifType=miranda) | 22 | miRNA: 3' ucuuaggaaCGGGCCCACGUaa 5' | -23.9 |
|  |  |  | \|:\|\|\|\|\|\|\|\|\| |  |
|  |  |  | Target:5' gcgcccgcgGTCCGGGTGCAgc 3' |  |
|  |  |  |  |  |
| hsa-miR-92b* | 218~239 | 22 | miRNA: 3' guGACGUGGCGCAGGGCAGGGa 5' | -23.3 |
|  |  |  | \|\| \|:\|: \| \|\|\|\|\|\|\|\|\| |  |
|  |  |  | Target:5' ccCTTCGCTCCCTCCCGTCCCc 3' |  |
|  |  |  |  |  |
| hsa-miR-1914* | [151~176](http://regrna.mbc.nctu.edu.tw/php/showtable.php?ColorRegion=151~176&FileDir=tmp/20161027/085354&SeqID=085354&MotifInfo=miRNA%20Target%20Sites&MotifType=miranda) | 26 | miRNA: 3' ggAGGGUCAC----GCCCUGGGGAGg 5' | -23 |
|  |  |  | \|\|:\| \|\|\| \| \| :\|\|\|\|\|\| |  |
|  |  |  | Target:5' ctTCTCTGTGCTCCCCGCGCCCCTCt 3' |  |
|  |  |  |  |  |
| hsa-miR-3190 | [397~420](http://regrna.mbc.nctu.edu.tw/php/showtable.php?ColorRegion=397~420&FileDir=tmp/20161027/085354&SeqID=085354&MotifInfo=miRNA%20Target%20Sites&MotifType=miranda) | 24 | miRNA: 3' agAGA--CCGGCAGAUGGAAGGUGu 5' | -21.8 |
|  |  |  | \|\|\| \|\|\|\| \| \|\|\|\|:\|\|\| |  |
|  |  |  | Target:5' tcTCTTCGGCC-CCCCCCTTTCACg 3' |  |
|  |  |  |  |  |
| hsa-miR-675 | [154~172](http://regrna.mbc.nctu.edu.tw/php/showtable.php?ColorRegion=154~172&FileDir=tmp/20161027/085354&SeqID=085354&MotifInfo=miRNA%20Target%20Sites&MotifType=miranda) | 19 | miRNA: 3' guGACACCCGGGAGAGGCGUGGu 5' | -21.6 |
|  |  |  | \|\|\|\|\| \|\|\| \|\|\|\|:\|\| |  |
|  |  |  | Target:5' ctCTGTG----CTCCCCGCGCCc 3' |  |
|  |  |  |  |  |
| hsa-miR-500a* | [67~88](http://regrna.mbc.nctu.edu.tw/php/showtable.php?ColorRegion=67~88&FileDir=tmp/20161027/085354&SeqID=085354&MotifInfo=miRNA%20Target%20Sites&MotifType=miranda) | 22 | miRNA: 3' gucuuaggaaCGGGUCCACGUa 5' | -21.4 |
|  |  |  | \|:\|\|:\|\|\|\|\|\| |  |
|  |  |  | Target:5' ggcgcccgcgGTCCGGGTGCAg 3' |  |
|  |  |  |  |  |
| hsa-miR-138 | [691~714](http://regrna.mbc.nctu.edu.tw/php/showtable.php?ColorRegion=691~714&FileDir=tmp/20161027/085354&SeqID=085354&MotifInfo=miRNA%20Target%20Sites&MotifType=miranda) | 24 | miRNA: 3' gcCGGACUAAGUGUUGUG-GUCGa 5' | -20 |
|  |  |  | \|:: \|:\|\|::\|:\|\|\|\| \|\|\|\| |  |
|  |  |  | Target:5' ctGTTCGGTTTGCGACACGCAGCa 3' |  |
|  |  |  |  |  |
| hsa-miR-323b-5p | [544~564](http://regrna.mbc.nctu.edu.tw/php/showtable.php?ColorRegion=544~564&FileDir=tmp/20161027/085354&SeqID=085354&MotifInfo=miRNA%20Target%20Sites&MotifType=miranda) | 21 | miRNA: 3' acGCUUGAGUGGUGCCUGUUGGa 5' | -19.9 |
|  |  |  | \|\| \|\|\| \|\| :\|\|\|\|\|\|:\| |  |
|  |  |  | Target:5' ccCGTCCTC-CC-TGGACAATCa 3' |  |
|  |  |  |  |  |
| hsa-miR-1306 | [670~687](http://regrna.mbc.nctu.edu.tw/php/showtable.php?ColorRegion=670~687&FileDir=tmp/20161027/085354&SeqID=085354&MotifInfo=miRNA%20Target%20Sites&MotifType=miranda) | 18 | miRNA: 3' gugGUGGUCUCGGUUGCa 5' | -19.7 |
|  |  |  | \|\| \|\|:\|\|\|\|\|\|\| |  |
|  |  |  | Target:5' cggCAGGAGGGCCAACGc 3' |  |
|  |  |  |  |  |
| hsa-miR-371-3p | [635~656](http://regrna.mbc.nctu.edu.tw/php/showtable.php?ColorRegion=635~656&FileDir=tmp/20161027/085354&SeqID=085354&MotifInfo=miRNA%20Target%20Sites&MotifType=miranda) | 22 | miRNA: 3' ugUGAGUU-UUCUACCGCCGUGAa 5' | -19.3 |
|  |  |  | \|\|\|\|:: \|\|\| \|\|\|\|\|:\|\| |  |
|  |  |  | Target:5' cgACTCGGCCAGA--GCGGCGCTg 3' |  |
|  |  |  |  |  |
| hsa-miR-654-5p | [510~529](http://regrna.mbc.nctu.edu.tw/php/showtable.php?ColorRegion=510~529&FileDir=tmp/20161027/085354&SeqID=085354&MotifInfo=miRNA%20Target%20Sites&MotifType=miranda) | 20 | miRNA: 3' cgUGUACAAGACGCCGGGUGGu 5' | -19.1 |
|  |  |  | \|\|\| \|\|\| \|\|\|\|\|:\|\| |  |
|  |  |  | Target:5' gtACAACATCT--GGCCCGCCc 3' |  |
|  |  |  |  |  |
| hsa-miR-377* | [72~93](http://regrna.mbc.nctu.edu.tw/php/showtable.php?ColorRegion=72~93&FileDir=tmp/20161027/085354&SeqID=085354&MotifInfo=miRNA%20Target%20Sites&MotifType=miranda) | 22 | miRNA: 3' cuuaaguGGUUCCCGUUGGAGa 5' | -18.7 |
|  |  |  | \|\|::\| \|\|\|:\|\|\|\| |  |
|  |  |  | Target:5' ccgcggtCCGGGTGCAGCCTCc 3' |  |
|  |  |  |  |  |
| hsa-miR-1233 | [26~47](http://regrna.mbc.nctu.edu.tw/php/showtable.php?ColorRegion=26~47&FileDir=tmp/20161027/085354&SeqID=085354&MotifInfo=miRNA%20Target%20Sites&MotifType=miranda) | 22 | miRNA: 3' gacGCCCUCCUG--UCCCGAGu 5' | -18.5 |
|  |  |  | \|\| \|\| \|:\| \|\|\|\|\|\|\| |  |
|  |  |  | Target:5' gctCGCGACGGCAGAGGGCTCc 3' |  |
|  |  |  |  |  |
| hsa-miR-3153 | [479~501](http://regrna.mbc.nctu.edu.tw/php/showtable.php?ColorRegion=479~501&FileDir=tmp/20161027/085354&SeqID=085354&MotifInfo=miRNA%20Target%20Sites&MotifType=miranda) | 23 | miRNA: 3' uuUACAGGGAUGAGCGAAAGGGg 5' | -17.9 |
|  |  |  | \|\| \|\|\|\| \|: \| :\|\|\|\|\|\| |  |
|  |  |  | Target:5' xxATTTCCCGATACCTTTTCCCc 3' |  |
|  |  |  |  |  |
| hsa-miR-3679-5p | [441~463](http://regrna.mbc.nctu.edu.tw/php/showtable.php?ColorRegion=441~463&FileDir=tmp/20161027/085354&SeqID=085354&MotifInfo=miRNA%20Target%20Sites&MotifType=miranda) | 23 | miRNA: 3' agGGGAAGGGACGGUAUAGGAGu 5' | -17.9 |
|  |  |  | \|:\|\| \|\|\| \|: \|\|\|\|\|\|: |  |
|  |  |  | Target:5' atCTCTGCCCCCCTCTATCCTTg 3' |  |
|  |  |  |  |  |
| hsa-miR-134 | [90~112](http://regrna.mbc.nctu.edu.tw/php/showtable.php?ColorRegion=90~112&FileDir=tmp/20161027/085354&SeqID=085354&MotifInfo=miRNA%20Target%20Sites&MotifType=miranda) | 23 | miRNA: 3' ggGGAGA-CCAGUUGGUCAGUGu 5' | -17.8 |
|  |  |  | \|\| \|\| \| \| \|\|\|\|\|\|\|\| |  |
|  |  |  | Target:5' ctCCACTCCGCCCCCCAGTCACc 3' |  |
|  |  |  |  |  |
| hsa-miR-4267 | [50~68](http://regrna.mbc.nctu.edu.tw/php/showtable.php?ColorRegion=50~68&FileDir=tmp/20161027/085354&SeqID=085354&MotifInfo=miRNA%20Target%20Sites&MotifType=miranda) | 21 | miRNA: 3' caCGG---UGG--CUCGACCu 5' | -17.7 |
|  |  |  | \|\|\| \|\|\| \|\|\|\|\|\|\| |  |
|  |  |  | Target:5' cgGCCCAAACCXXGAGCTGGg 3' |  |
|  |  |  |  |  |
| hsa-miR-4297 | [667~682](http://regrna.mbc.nctu.edu.tw/php/showtable.php?ColorRegion=667~682&FileDir=tmp/20161027/085354&SeqID=085354&MotifInfo=miRNA%20Target%20Sites&MotifType=miranda) | 16 | miRNA: 3' guGUCUGUCCUUCCGu 5' | -17.4 |
|  |  |  | \| \|:\|\|\|\|\|:\|\|\| |  |
|  |  |  | Target:5' gtCCGGCAGGAGGGCc 3' |  |
|  |  |  |  |  |
| hsa-miR-3925 | [328~349](http://regrna.mbc.nctu.edu.tw/php/showtable.php?ColorRegion=328~349&FileDir=tmp/20161027/085354&SeqID=085354&MotifInfo=miRNA%20Target%20Sites&MotifType=miranda) | 22 | miRNA: 3' uccGAGGUGAAAGUCAAGAGAa 5' | -17.1 |
|  |  |  | \|\|\|: \| \| \| \|\|\|\|\|\|\| |  |
|  |  |  | Target:5' tttCTCTCCGTGCTGTTCTCTc 3' |  |
|  |  |  |  |  |
| hsa-miR-3124 | [613~638](http://regrna.mbc.nctu.edu.tw/php/showtable.php?ColorRegion=613~638&FileDir=tmp/20161027/085354&SeqID=085354&MotifInfo=miRNA%20Target%20Sites&MotifType=miranda) | 26 | miRNA: 3' cuGAAACG-G-AAGC---GGGCGCUu 5' | -16.9 |
|  |  |  | \| \| \|\| \| \|\|\|\| \|\|\|\|\|\|\| |  |
|  |  |  | Target:5' ccCGTCGCACATTCGGCCCCCGCGAc 3' |  |
|  |  |  |  |  |
| hsa-miR-1237 | [26~44](http://regrna.mbc.nctu.edu.tw/php/showtable.php?ColorRegion=26~44&FileDir=tmp/20161027/085354&SeqID=085354&MotifInfo=miRNA%20Target%20Sites&MotifType=miranda) | 19 | miRNA: 3' gaccCCCUGCCUCGUCUUCCu 5' | -16.6 |
|  |  |  | \| \|\|\|\| \|\|\|\|\|:\|\| |  |
|  |  |  | Target:5' gctcGCGACG--GCAGAGGGc 3' |  |
|  |  |  |  |  |
| hsa-miR-3184 | [149~176](http://regrna.mbc.nctu.edu.tw/php/showtable.php?ColorRegion=149~176&FileDir=tmp/20161027/085354&SeqID=085354&MotifInfo=miRNA%20Target%20Sites&MotifType=miranda) | 28 | miRNA: 3' uuuucGAGCCA-GA---CUCCGGGGAGu 5' | -16.1 |
|  |  |  | \|\|\| \|\| \|\| : \|\|\|\|\|\|\| |  |
|  |  |  | Target:5' cgcttCTCTGTGCTCCCCGCGCCCCTCt 3' |  |
|  |  |  |  |  |
| hsa-miR-1299 | [727~748](http://regrna.mbc.nctu.edu.tw/php/showtable.php?ColorRegion=727~748&FileDir=tmp/20161027/085354&SeqID=085354&MotifInfo=miRNA%20Target%20Sites&MotifType=miranda) | 22 | miRNA: 3' agggaguGUGUCUUAAGGUCUu 5' | -15.9 |
|  |  |  | \|:\| \|: \|\|\|\|\|\|\| |  |
|  |  |  | Target:5' gcagcgtCGCCGGCTTCCAGAc 3' |  |
|  |  |  |  |  |
| hsa-miR-1321 | [537~556](http://regrna.mbc.nctu.edu.tw/php/showtable.php?ColorRegion=537~556&FileDir=tmp/20161027/085354&SeqID=085354&MotifInfo=miRNA%20Target%20Sites&MotifType=miranda) | 15 | miRNA: 3' uaGUGUAAGUGGAGGGAc 5' | -15.9 |
|  |  |  | \| \|:\| \|\|\|\|\|\|\| |  |
|  |  |  | Target:5' agCCCGT---CCTCCCTg 3' |  |
|  |  |  |  |  |
| hsa-let-7d | [425~446](http://regrna.mbc.nctu.edu.tw/php/showtable.php?ColorRegion=425~446&FileDir=tmp/20161027/085354&SeqID=085354&MotifInfo=miRNA%20Target%20Sites&MotifType=miranda) | 22 | miRNA: 3' uuGAUACGUUGGAUGAUGGAGa 5' | -15.8 |
|  |  |  | \|\|:\| : \|\| \|\|\|\|:\|\|\| |  |
|  |  |  | Target:5' ctCTGTCTCTCCCACTATCTCt 3' |  |
|  |  |  |  |  |
| hsa-miR-3191 | [217~240](http://regrna.mbc.nctu.edu.tw/php/showtable.php?ColorRegion=217~240&FileDir=tmp/20161027/085354&SeqID=085354&MotifInfo=miRNA%20Target%20Sites&MotifType=miranda) | 24 | miRNA: 3' gacaGACCGGUCGA-UGCAGGGGu 5' | -15.2 |
|  |  |  | :\| \|\|: \|\| \|\|\|\|\|\|\| |  |
|  |  |  | Target:5' tcccTTCGCTCCCTCCCGTCCCCc 3' |  |
|  |  |  |  |  |
| hsa-miR-3940 | [8~29](http://regrna.mbc.nctu.edu.tw/php/showtable.php?ColorRegion=8~29&FileDir=tmp/20161027/085354&SeqID=085354&MotifInfo=miRNA%20Target%20Sites&MotifType=miranda) | 22 | miRNA: 3' uucacccGACCCUAGGCCCGAc 5' | -15 |
|  |  |  | \| \| \|: \|\|\|\|\|\|\| |  |
|  |  |  | Target:5' ccccctcCCGAGGCCCGGGCTc 3' |  |
|  |  |  |  |  |
| hsa-miR-4306 | [335~351](http://regrna.mbc.nctu.edu.tw/php/showtable.php?ColorRegion=335~351&FileDir=tmp/20161027/085354&SeqID=085354&MotifInfo=miRNA%20Target%20Sites&MotifType=miranda) | 17 | miRNA: 3' augACGGAAAGAGAGGu 5' | -15 |
|  |  |  | \|\|\|: \|\|\|\|\|\|\|\| |  |
|  |  |  | Target:5' ccgTGCTGTTCTCTCCc 3' |  |
|  |  |  |  |  |
| hsa-miR-3687 | [673~699](http://regrna.mbc.nctu.edu.tw/php/showtable.php?ColorRegion=673~699&FileDir=tmp/20161027/085354&SeqID=085354&MotifInfo=miRNA%20Target%20Sites&MotifType=miranda) | 25 | miRNA: 3' ugcAGCGU-GCUUGCGGACAGGCCc 5' | -14.7 |
|  |  |  | \| \|\|\| \| \| \|\|\|\|\|\|\| |  |
|  |  |  | Target:5' cgcTGGCAGXXXAGGAGTGTCCGGc 3' |  |
|  |  |  |  |  |
| hsa-miR-425* | [470~490](http://regrna.mbc.nctu.edu.tw/php/showtable.php?ColorRegion=470~490&FileDir=tmp/20161027/085354&SeqID=085354&MotifInfo=miRNA%20Target%20Sites&MotifType=miranda) | 24 | miRNA: 3' ccCGCCU-GUGCUGU-AAGGGCUa 5' | -14.44 |
|  |  |  | \|\| \|\| \| \| \| \|\|\|\|\|\|\| |  |
|  |  |  | Target:5' caGCTGACCTCXXXATTTCCCGAt 3' |  |
|  |  |  |  |  |
| hsa-miR-483-3p | [648~668](http://regrna.mbc.nctu.edu.tw/php/showtable.php?ColorRegion=648~668&FileDir=tmp/20161027/085354&SeqID=085354&MotifInfo=miRNA%20Target%20Sites&MotifType=miranda) | 21 | miRNA: 3' uuCUGCCCUCCUCUCCUCACu 5' | -14.3 |
|  |  |  | \| :\|\| \|\| \|\|\|\|\|\|\| |  |
|  |  |  | Target:5' gcGCTGGCAGXXXAGGAGTGt 3' |  |
|  |  |  |  |  |
| hsa-let-7a | [425~446](http://regrna.mbc.nctu.edu.tw/php/showtable.php?ColorRegion=425~446&FileDir=tmp/20161027/085354&SeqID=085354&MotifInfo=miRNA%20Target%20Sites&MotifType=miranda) | 22 | miRNA: 3' uuGAUAUGUUGGAUGAUGGAGu 5' | -14.2 |
|  |  |  | \|\|:\| : \|\| \|\|\|\|:\|\|\| |  |
|  |  |  | Target:5' ctCTGTCTCTCCCACTATCTCt 3' |  |
|  |  |  |  |  |
| hsa-miR-219-5p | [544~564](http://regrna.mbc.nctu.edu.tw/php/showtable.php?ColorRegion=544~564&FileDir=tmp/20161027/085354&SeqID=085354&MotifInfo=miRNA%20Target%20Sites&MotifType=miranda) | 21 | miRNA: 3' ucuuaacgcaaACCUGUUAGu 5' | -13.7 |
|  |  |  | \|\|\|\|\|\|\|\|\| |  |
|  |  |  | Target:5' cccgtcctcccTGGACAATCa 3' |  |
|  |  |  |  |  |
| hsa-let-7b | 420~446 | 27 | miRNA: 3' uuGGUGUGUUGGA-----UGAUGGAGu 5' | -13.5 |
|  |  |  | :\|\|\| \| ::\|\| \|\|\|\|:\|\|\| |  |
|  |  |  | Target:5' xtTCACTCTGTCTCTCCCACTATCTCt 3' |  |
|  |  |  |  |  |
| hsa-miR-3613-5p | [496~517](http://regrna.mbc.nctu.edu.tw/php/showtable.php?ColorRegion=496~517&FileDir=tmp/20161027/085354&SeqID=085354&MotifInfo=miRNA%20Target%20Sites&MotifType=miranda) | 22 | miRNA: 3' cuuguuuuuUUUUUCAUGUUGu 5' | -13.4 |
|  |  |  | :\|\|\|\|\|\|\|\|\|\|\| |  |
|  |  |  | Target:5' ttcccccccGAAAAGTACAACa 3' |  |
|  |  |  |  |  |
| hsa-miR-625 | [484~503](http://regrna.mbc.nctu.edu.tw/php/showtable.php?ColorRegion=484~503&FileDir=tmp/20161027/085354&SeqID=085354&MotifInfo=miRNA%20Target%20Sites&MotifType=miranda) | 20 | miRNA: 3' ccuGAUAUCUUGAAAGGGGGa 5' | -13.3 |
|  |  |  | \| \|\|\| :\|\|\|\|\|\|\|\| |  |
|  |  |  | Target:5' tccCGATA-CCTTTTCCCCCc 3' |  |
|  |  |  |  |  |
| hsa-miR-1276 | [180~205](http://regrna.mbc.nctu.edu.tw/php/showtable.php?ColorRegion=180~205&FileDir=tmp/20161027/085354&SeqID=085354&MotifInfo=miRNA%20Target%20Sites&MotifType=miranda) | 26 | miRNA: 3' acAGA--GGUGUC----CCGAGAAAu 5' | -13.1 |
|  |  |  | \|\|\| \|\| \|:\| \|\|\|\|\|\|\| |  |
|  |  |  | Target:5' xgTCTGGCCCCGGCCCCCGCTCTTTc 3' |  |
|  |  |  |  |  |
| hsa-miR-3202 | [125~146](http://regrna.mbc.nctu.edu.tw/php/showtable.php?ColorRegion=125~146&FileDir=tmp/20161027/085354&SeqID=085354&MotifInfo=miRNA%20Target%20Sites&MotifType=miranda) | 22 | miRNA: 3' uaauuucgagaaGAGGGAAGGu 5' | -12.9 |
|  |  |  | \| \|\|\|\|\|\|\| |  |
|  |  |  | Target:5' ccctcgacgtggCGCCCTTCCc 3' |  |
|  |  |  |  |  |
| hsa-miR-3119 | [581~598](http://regrna.mbc.nctu.edu.tw/php/showtable.php?ColorRegion=581~598&FileDir=tmp/20161027/085354&SeqID=085354&MotifInfo=miRNA%20Target%20Sites&MotifType=miranda) | 18 | miRNA: 3' cgguaGUUUCAAUUUUCGGu 5' | -12.1 |
|  |  |  | \|\|\|\| \|\|\|\|\|\|\| |  |
|  |  |  | Target:5' cccccCAAA--AAAAAGCCa 3' |  |
|  |  |  |  |  |
| hsa-let-7g | [425~446](http://regrna.mbc.nctu.edu.tw/php/showtable.php?ColorRegion=425~446&FileDir=tmp/20161027/085354&SeqID=085354&MotifInfo=miRNA%20Target%20Sites&MotifType=miranda) | 22 | miRNA: 3' uuGACAUGUUUGAUGAUGGAGu 5' | -11.94 |
|  |  |  | \|\|\|\| : \| \|\|\|\|:\|\|\| |  |
|  |  |  | Target:5' ctCTGTCTCTCCCACTATCTCt 3' |  |
|  |  |  |  |  |
| hsa-let-7c | [425~446](http://regrna.mbc.nctu.edu.tw/php/showtable.php?ColorRegion=425~446&FileDir=tmp/20161027/085354&SeqID=085354&MotifInfo=miRNA%20Target%20Sites&MotifType=miranda) | 22 | miRNA: 3' uuGGUAUGUUGGAUGAUGGAGu 5' | -11.7 |
|  |  |  | \|::\| : \|\| \|\|\|\|:\|\|\| |  |
|  |  |  | Target:5' ctCTGTCTCTCCCACTATCTCt 3' |  |
|  |  |  |  |  |
| hsa-miR-3919 | [138~159](http://regrna.mbc.nctu.edu.tw/php/showtable.php?ColorRegion=138~159&FileDir=tmp/20161027/085354&SeqID=085354&MotifInfo=miRNA%20Target%20Sites&MotifType=miranda) | 22 | miRNA: 3' ugacucaGGAAAC-AAGAGACg 5' | -11.2 |
|  |  |  | \|\|\| \| \|\|\|\|\|\|\| |  |
|  |  |  | Target:5' gcccttcCCTCCGCTTCTCTGt 3' |  |
|  |  |  |  |  |
| has-miR-101 | [324~344](http://regrna.mbc.nctu.edu.tw/php/showtable.php?ColorRegion=324~344&FileDir=tmp/20161027/085354&SeqID=085354&MotifInfo=miRNA%20Target%20Sites&MotifType=miranda) | 21 | miRNA: 3' aagucAAUAGUGUCAUGACAu 5' | -11.1 |
|  |  |  | \|\| \|\| \| \|\|:\|\|\|\| |  |
|  |  |  | Target:5' tctgtTTCTCTCCGTGCTGTt 3' |  |
|  |  |  |  |  |
| hsa-miR-759 | [411~430](http://regrna.mbc.nctu.edu.tw/php/showtable.php?ColorRegion=411~430&FileDir=tmp/20161027/085354&SeqID=085354&MotifInfo=miRNA%20Target%20Sites&MotifType=miranda) | 22 | miRNA: 3' caguuuuaacaaACGUGAGACg 5' | -10.7 |
|  |  |  | \| \|\|\|\|\|\|\| |  |
|  |  |  | Target:5' cctttcacgxxxTTCACTCTGt 3' |  |
|  |  |  |  |  |
| hsa-miR-3916 | [307~335](http://regrna.mbc.nctu.edu.tw/php/showtable.php?ColorRegion=307~335&FileDir=tmp/20161027/085354&SeqID=085354&MotifInfo=miRNA%20Target%20Sites&MotifType=miranda) | 29 | miRNA: 3' gacucUUGGUCGGU-AAAG--AAGGAGAa 5' | -10.3 |
|  |  |  | \|\|::\| \|: \|\|\|\| \|\|:\|\|\|\| |  |
|  |  |  | Target:5' ggattAATTACACGCTTTCTGTTTCTCTc 3' |  |
|  |  |  |  |  |
| hsa-miR-135a | [578~600](http://regrna.mbc.nctu.edu.tw/php/showtable.php?ColorRegion=578~600&FileDir=tmp/20161027/085354&SeqID=085354&MotifInfo=miRNA%20Target%20Sites&MotifType=miranda) | 23 | miRNA: 3' aguguauccUUAUUUUUCGGUAu 5' | -9.7 |
|  |  |  | \|\| \|\|\|\|\|\|\|\|\|\| |  |
|  |  |  | Target:5' cccccccccAAAAAAAAGCCATc 3' |  |
|  |  |  |  |  |
| hsa-miR-548m | [471~497](http://regrna.mbc.nctu.edu.tw/php/showtable.php?ColorRegion=471~497&FileDir=tmp/20161027/085354&SeqID=085354&MotifInfo=miRNA%20Target%20Sites&MotifType=miranda) | 21 | miRNA: 3' guuuUUGGUGUUUAUGGAAAc 5' | -9.1 |
|  |  |  | \| :: \| :\|\|\|\|\|\|\|\| |  |
|  |  |  | Target:5' cxxxATTTCCCGATACCTTTt 3' |  |
|  |  |  |  |  |
| hsa-miR-223 | [451~478](http://regrna.mbc.nctu.edu.tw/php/showtable.php?ColorRegion=451~478&FileDir=tmp/20161027/085354&SeqID=085354&MotifInfo=miRNA%20Target%20Sites&MotifType=miranda) | 28 | miRNA: 3' accccAUA--AACUGU----UUGACUGu 5' | -9 |
|  |  |  | \|\|\| \|\|\|\|:\| \|:\|\|\|\|\| |  |
|  |  |  | Target:5' ccctcTATCCTTGATACAACAGCTGACc 3' |  |
|  |  |  |  |  |
| hsa-miR-3118 | [90~112](http://regrna.mbc.nctu.edu.tw/php/showtable.php?ColorRegion=90~112&FileDir=tmp/20161027/085354&SeqID=085354&MotifInfo=miRNA%20Target%20Sites&MotifType=miranda) | 23 | miRNA: 3' ucuuaaaaguauuacGUCAGUGu 5' | -8.8 |
|  |  |  | \|\|\|\|\|\|\| |  |
|  |  |  | Target:5' ctccactccgcccccCAGTCACc 3' |  |
|  |  |  |  |  |
| hsa-miR-135b | [578~600](http://regrna.mbc.nctu.edu.tw/php/showtable.php?ColorRegion=578~600&FileDir=tmp/20161027/085354&SeqID=085354&MotifInfo=miRNA%20Target%20Sites&MotifType=miranda) | 23 | miRNA: 3' aguguauccUUACUUUUCGGUAu 5' | -8.6 |
|  |  |  | \|\| \|\|\|\|\|\|\|\|\| |  |
|  |  |  | Target:5' cccccccccAAAAAAAAGCCATc 3' |  |
|  |  |  |  |  |
| hsa-miR-488 | [399~419](http://regrna.mbc.nctu.edu.tw/php/showtable.php?ColorRegion=399~419&FileDir=tmp/20161027/085354&SeqID=085354&MotifInfo=miRNA%20Target%20Sites&MotifType=miranda) | 21 | miRNA: 3' cugguucuuuaucGGAAAGUu 5' | -7.42 |
|  |  |  | \|\|\|\|\|\|\| |  |
|  |  |  | Target:5' tcttcggccccccCCTTTCAc 3' |  |
|  |  |  |  |  |
